# New evidence of megafaunal bone damage indicates late colonization of Madagascar

**DOI:** 10.1371/journal.pone.0204368

**Published:** 2018-10-10

**Authors:** Atholl Anderson, Geoffrey Clark, Simon Haberle, Tom Higham, Malgosia Nowak-Kemp, Amy Prendergast, Chantal Radimilahy, Lucien M. Rakotozafy, Ramilisonina, Jean-Luc Schwenninger, Malika Virah-Sawmy, Aaron Camens

**Affiliations:** 1 Department of Archaeology and Natural History, Australian National University, Canberra ACT Australia; 2 ORAU, Research Laboratory for Archaeology, Oxford, United Kingdom; 3 University Museum of Natural History, Oxford, United Kingdom; 4 School of Geography, University of Melbourne, Melbourne VIC, Australia; 5 Musée d’Art et d’Archaéologie, The University of Antananarivo, Antananarivo BP, Isoraka, Madagascar; 6 Plant Conservation Unit, Department of Biological Sciences, University of Cape Town, Cape Town, South Africa; 7 Ecology and Evolution Group, College of Science and Engineering, Flinders University, Adelaide SA, Australia; University of Otago, NEW ZEALAND

## Abstract

The estimated period in which human colonization of Madagascar began has expanded recently to 5000–1000 y B.P., six times its range in 1990, prompting revised thinking about early migration sources, routes, maritime capability and environmental changes. Cited evidence of colonization age includes anthropogenic palaeoecological data 2500–2000 y B.P., megafaunal butchery marks 4200–1900 y B.P. and OSL dating to 4400 y B.P. of the Lakaton’i Anja occupation site. Using large samples of newly-excavated bone from sites in which megafaunal butchery was earlier dated >2000 y B.P. we find no butchery marks until ~1200 y B.P., with associated sedimentary and palynological data of initial human impact about the same time. Close analysis of the Lakaton’i Anja chronology suggests the site dates <1500 y B.P. Diverse evidence from bone damage, palaeoecology, genomic and linguistic history, archaeology, introduced biota and seafaring capability indicate initial human colonization of Madagascar 1350–1100 y B.P.

## Introduction

**Fig 1:**
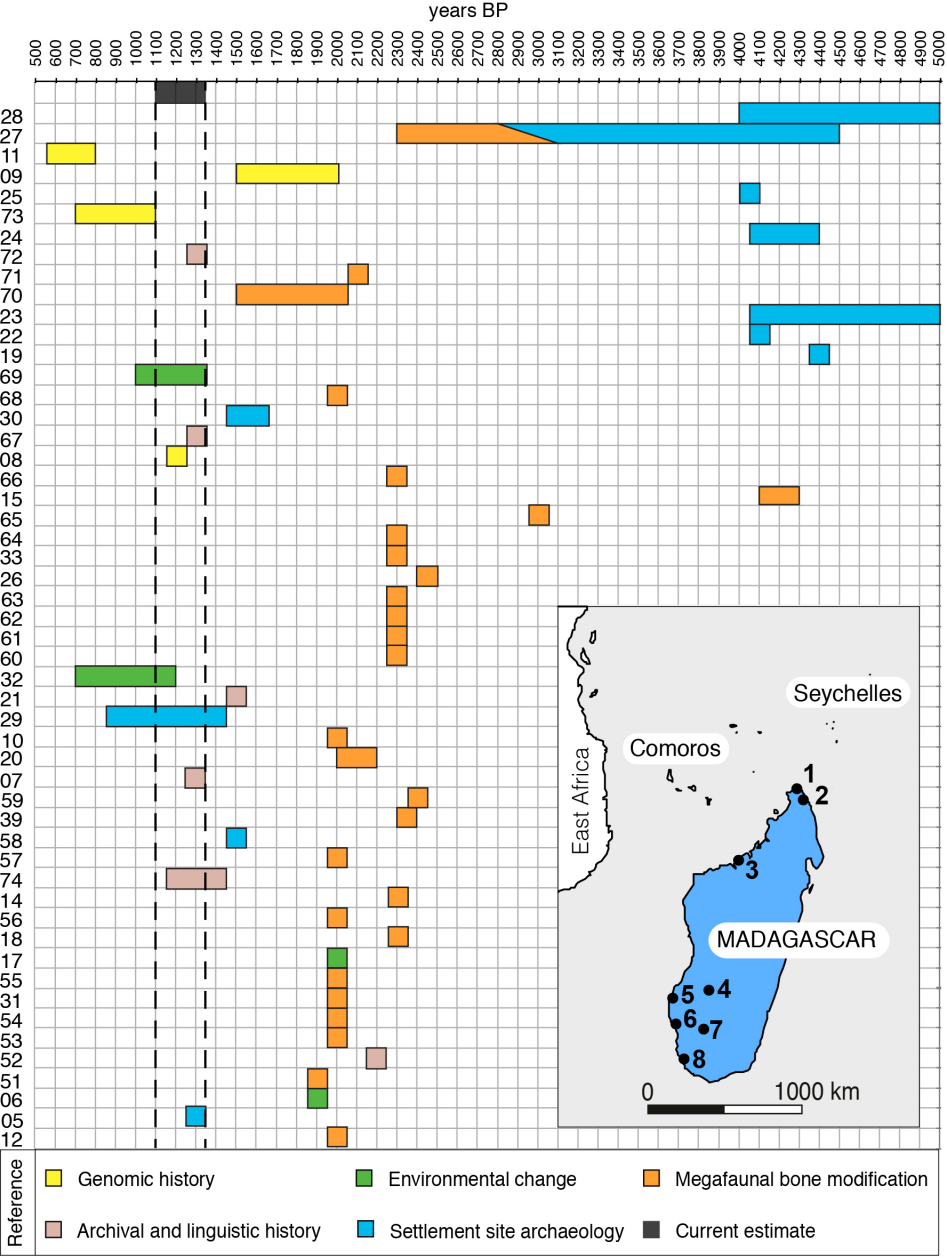
Chronological ranges of IHCE (see text) for Madagascar shown by main source of evidence and in publication date order bottom to top (y axis). Numbers = text references. Inset: Madagascar with sites discussed in text; 1 = Lakaton’i Anja, 2 = Ambohiposa, 3 = Anjohibe, 4 = Tsirave, 5 = Lamboharana, 6 = Ambolisatra, 7 = Taolambiby, 8 = Itampolo.

In the “jigsaw puzzle of Indian Ocean prehistory” [1] the most difficult piece to fit is Madagascar, the world’s largest oceanic island and tacitly accepted as key to understanding how prehistoric colonization developed across the western Indian Ocean [2]. By 1990, evidence from maritime history, linguistics and archaeology indicated settlement of Madagascar in the range 2000–1350 y B.P. [3–7]. Since then, especially since 2011, the range of initial human colonization estimates (IHCE) has increased six-fold to 5000–1000 y B.P. (Fig 1). There are IHCE of ~1200 to 950–550 y B.P. from genomic histories [8–11] but most expansion has come from ^14^C dated megafaunal bones bearing damage interpreted as butchery 4200–1900 y B.P. [12–15]; palaeoecological analysis of sediment cores indicating anthropogenic changes 2200–1500 y B.P. [16–18]; and optically stimulated luminescence (OSL) dating of natural sediments containing some cultural remains at Lakaton’i Anja rockshelter to 4400–2200 y B.P. [19]. These IHCE have been adopted by archaeological hypotheses that envisage mid-Holocene (Later Stone Age) migration to Madagascar from East or South Africa and trans-oceanic voyaging from Neolithic Southeast Asia by 2500 y B.P. [1, 19–25]. Protracted human association with extinct megafauna is proposed accordingly [15, 18, 26–28]. Early fleeting colonization followed considerably later by lasting habitation, or early and continuing settlement that was low-density and cryptic until late florescence, are the implicit settlement models. Yet concerns exist about the provenance, age and modification of megafaunal bones [29–31], agencies of environmental change [28, 32] and interpretation of OSL results [2, 24]. In addition, inferring colonization up to 5000 y B.P. from these indirect evidential sources only emphasizes a chronological incongruity with direct archaeological sources in which no indubitable occupation sites in Madagascar are ^14^C dated earlier than ~1500 y B.P. [22, 24]. The question is whether IHCE on such direct evidence are plausibly eclipsed by those on indirect sources, as is now so widely assumed.

Here, we use evidence from new excavations of key sites in southwest Madagascar to critically examine the data and interpretations of indirect sources of colonization age in Madagascar. Large samples of megafaunal bone and associated AMS ^14^C dates from three sites are used to evaluate IHCE based on megafaunal bone modification, the most frequently cited source (53% of IHCE in Fig 1), and bone samples with purported cutmarks from older collections are re-examined (S1 File). A new sedimentary core helps elucidate the initiation of anthropogenic palaeoecological impact and an alternative interpretation is proposed of the Lakaton’i Anja chronology. The thrust of these analyses is towards a much younger IHCE range for Madagascar.

## Materials and methods

### Bone damage analysis

In experimental research, at least, three broad, overlapping categories of damage types can be recognized. I. Cultural cutmarks made by sharp stone or metal implements which are characteristically manifested as relatively deep and narrow, and v- or √-shaped in cross-section with crisply defined kerf walls [33, 34, 35]; II. Mechanical abrasion marks from movement between bone and coarse-grained sediment, the result of agencies such as trampling, mass movement, or fluvial action. Damage appears as numerous fine, shallow, short striations lacking chatter marks or regular patterns of orientation and anatomical location, and also as shallow, broad, and curving or irregular scoring, according to the sediment type and pressure involved [36, 37, 38]. III. Biological damage by predators and scavengers, which includes crushing, tooth-scoring, dents and holes from biting, and marks of animal gnawing [33, 39, 40]. There are many other kinds of taphonomic damage to bone surfaces; e.g., bone fractures, cortical lifting from sunlight exposure, and solution channels (etching) caused by root acids.

Interpreting bone damage involves serious, unresolved difficulties [40–42], which have been mitigated to some extent here by using large, whole (unsorted) assemblages of bone in which the original bone damage (that which occurred in depositional contexts prior to excavation) was recorded at the point of bone recovery, and also by assessing the in-site taphonomic circumstances, cultural context (if any), and the relative extent of damage morphologies. Sediments from excavations at Ambolisatra (4 m^2^), Taolambiby (14 m^2^) and Itampolo (5 m^2^) were wet-sieved through 2 mm mesh and recovered bone was partly sun-dried and examined in hand specimen for signs of damage (Figs K-N in S1 File). All bone material was retained but specimens with particular damage were bagged and returned to The Australian National University (ANU) in Canberra and stored in the Quarantine Laboratory in the Department of Archaeology and Natural History (ANH) in the College of Asia and the Pacific where they can be accessed. Field work permission was obtained through a Inter-University Agreement between The University of Antananarivo Musée d’Art et d’Archaéologie (ICMAA) and ANU-ANH (dated 31 May 2011). Bone was initially examined with a X10 light microscope. All possible cutmarks, examples of damage morphologies, and all damage otherwise difficult to characterize, were imaged using a Quanta 450 SEM under low vacuum and/or a MZ16 stereoscopic microscope, the latter providing three-dimensional images for cross-sectional profiles. An experimental protocol that successfully discriminated trampling from cutmarks [38] was employed indicatively.

Extinct lemur bones on which butchery damage had been perceived [14] in the 1911 Methuen collection (Oxford University Museum of Natural History), were examined in the same way. Thick curatorial wax coating these bones, which had frustrated earlier research on damage marks [13, 14], was removed with acetone before our cross-sectional profiles were obtained. Collections of megafaunal bone in the Grandidier collection, Muséum National d'Histoire Naturelle (MNHN, Paris), were examined under hand lens to estimate the nature and rates of cutmarking (S1 File).

### Radiocarbon dating

A total of 69 samples of bone (n = 65) and charcoal (n-4) were submitted to ORAU for AMS dating in 2011–2012 from our excavations at Ambolisatra, Itampolo and Taolambiby and bone from the Methuen collection (OUM). Samples were prepared using current pretreatment for bone and charcoal samples and calibrated using CALIB 7.1.0 with the SH Calibration curve (S1 File) with date ranges at two sigma (95.4%).

### Sedimentary coring

A Russian d-section corer was used to extract a 3 m core in 0.5 m sections from a saline coastal basin at Ambolisatra in 2011. The core site was approximately 2 m west of the Main Pit excavation (Fig M in S1 File). The cores were transported to the ANU and subsampled at 1 cm contiguous intervals for charcoal analysis. The pollen, spore and charcoal data were plotted in a pollen diagram against age using psimpoll 4.25 to describe zone boundaries representing statistically significant thresholds of change (Fig I in S1 File). Age results for the Ambolisatra sediments are reported in Table F in S1 File.

## Results

### Analysis of potentially cultural bone damage

In three categories of bone modification agency; cultural (mostly ‘cutmarking’), and mechanical and biological (glossed together here as ‘taphonomic’), some damage morphologies are recognizable but many overlap between categories [3, 33–42]. Agent diversity and morphological overlap raise concerns about intractable equifinality and consequent subjectivity in interpreting bone damage [40, 43], especially where there are small sample sizes and limited contextual data. In Madagascar, megafaunal bone analysis has focused selectively upon cultural interpretation of damage morphology [12–15] in samples from museum collections. Apparent cut marks have been taken as evidence of butchery [12–16, 18] and in the largest study [13, 14] as “definitive” evidence. As butchery is by definition perimortem, that conclusion has the merit that human occupation is dated by the age of the bone, a connection that is otherwise problematic for cultural bone modification. Perceived butchery marks on megafaunal bones dating 2300–2000 y B.P. were the mainstay of Madagascan IHCE, 1991–2011 (Fig 1).

**Fig 2:**
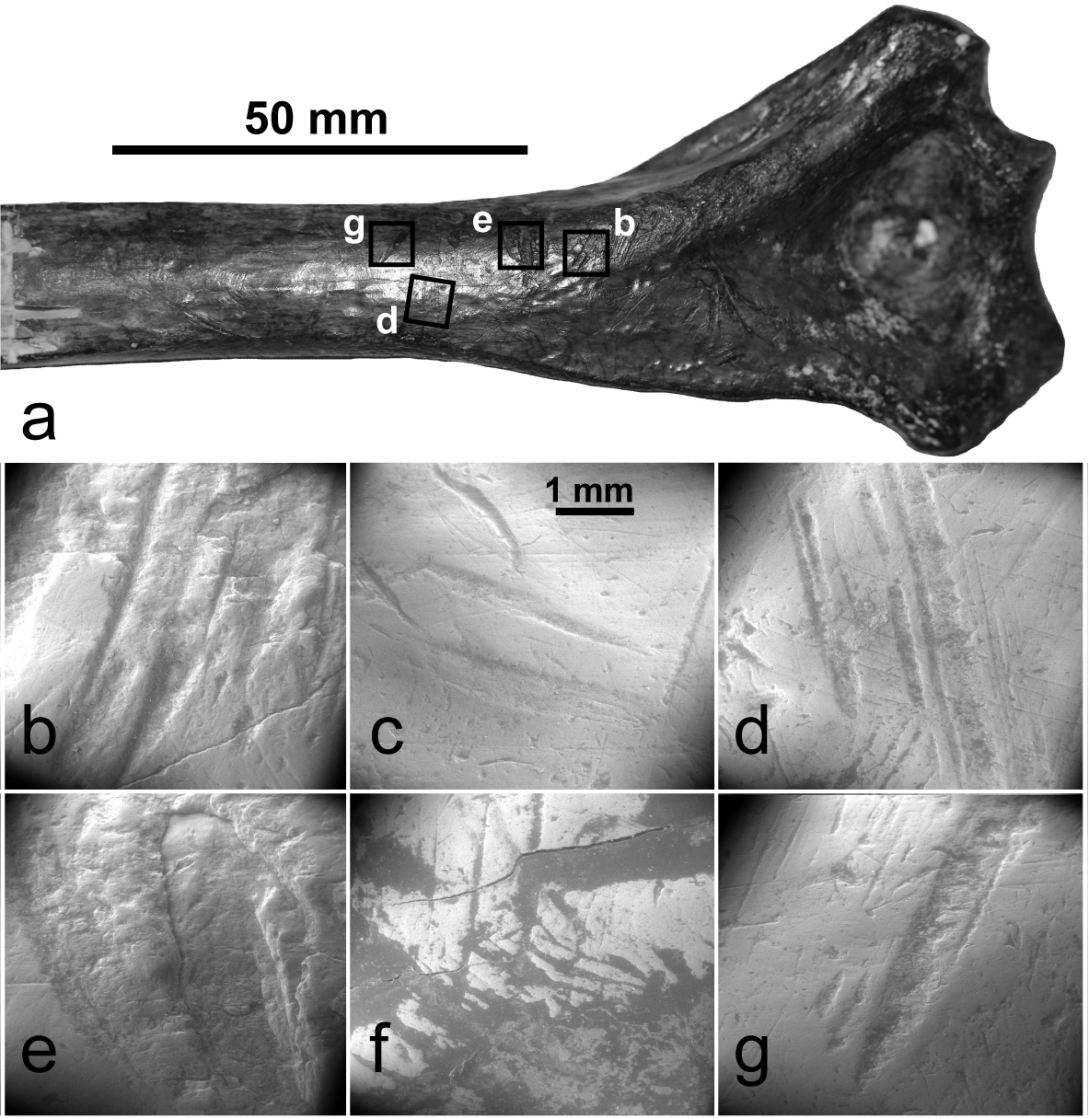
Re-examination of purported cutmarks [14] on *Palaeopropithecus ingens* distal humerus (OUM14342A) from Taolambiby: (a) Location of SEM images (locations c, f, on bone reverse); b-g SEM images indicating damage was by abrasive contact.

However, all of the Madagascan bones involved are from fundamentally palaeontological deposits that formed around waterholes or within cave systems and archaeological remains, where they occur at all, are scarce and surficial [29–31]; thus butchery seems improbable *a priori*. It is an inference largely by default, arising from limited consideration of taphonomic agencies in the published studies [12–15, 18] and a concomitant failure to recognize taphonomic damage in the analysed material [13–15]. To some extent that was unavoidable because 18 of the 21 purportedly butchered bones were collected AD 1898–1930, and they lacked documentation of spatial and stratigraphic context, recovery methods, sample selection, curation or other data relevant to taphonomic assessment. Re-analysis of 10 of these bones, exemplified by the most modified of them (Fig 2) did not indicate cutmarks, much less butchery (S1 File).

Our approach to Madagascan megafaunal bone damage assumes that most of it will have natural origins, because of the diversity of agencies involved and their potentially continual activity between original deposition and recent bone recovery. A null hypothesis that bone damage is taphonomic is open to rejection by finding original cutmark morphologies and cutmark repetition and by independent evidence of a cultural context. Our method compares damage morphologies at a microscopic level against a range of cultural and taphonomic interpretations, observes significant taphonomic features of the depositional environments, and measures the relative extent of cultural damage between extinct and extant faunas. This configurational approach [33] required large samples of megafaunal bone for which modification at the point of field recovery was known.

Excavations in 2011 at the three surviving subfossil sites in southwest Madagascar that had provided 55% of the modified megafaunal bones described in earlier research fulfilled these conditions. The recovered bone assemblage from Ambolisatra, Itampolo and Taolambiby (Fig 1) totals NISP = 2710, MNI = 110 (Table D in S1 File), of which megafaunal bone is NISP = 1787, MNI = 77. The main megafaunal taxa in order of abundance are: hippopotamus, crocodile, giant tortoise, giant lemurs, elephant birds. This sample is 42 times the size of the total sample (n = 43) of megafaunal bones previously analysed for cutmarks [14 and S1 File], and it enables calculation of robust rates of bone modification and comparison with previous data from the same sites.

**Fig 3:**
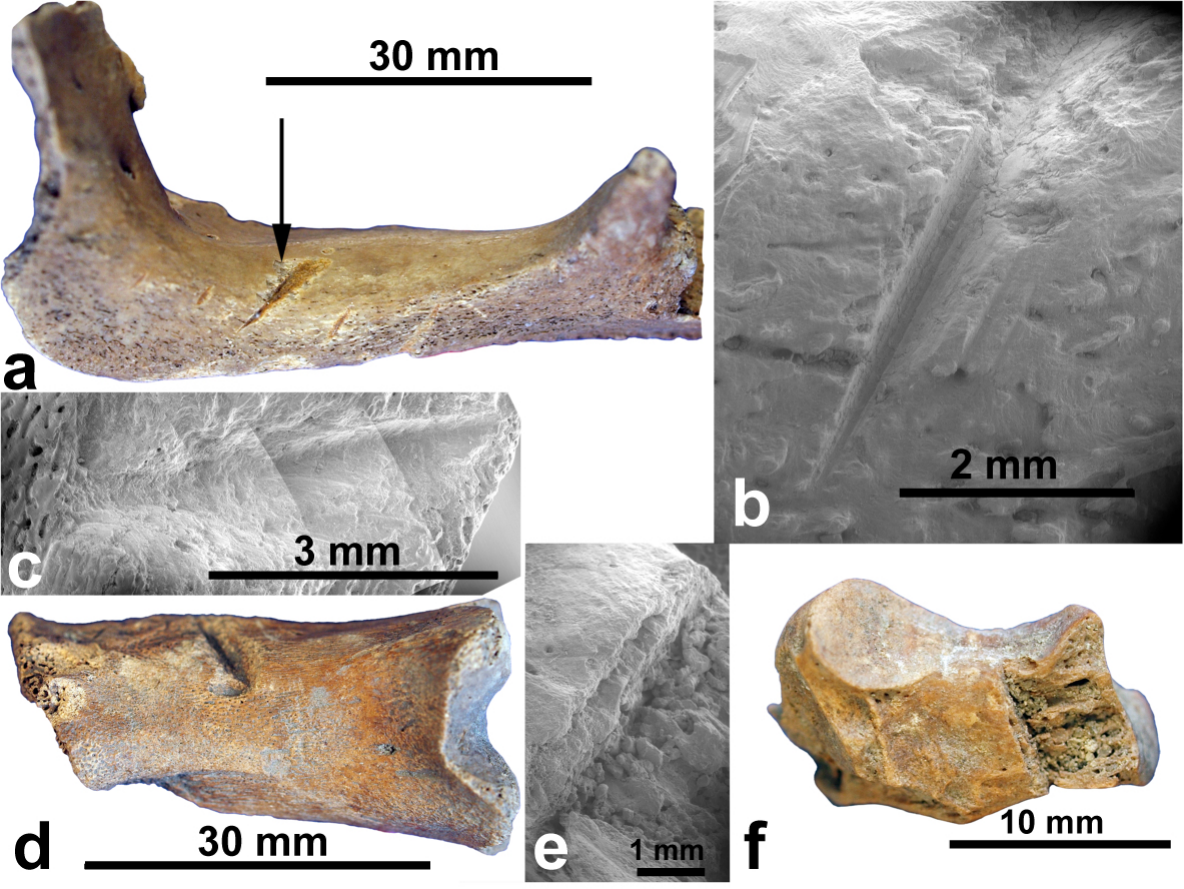
Possible cutmarks on newly excavated megafaunal bone: *Hippopotamus* jugal (ANU 107–1) from Itampolo, with cutmark under normal light (a) and SEM (b); similarly, metapodial of juvenile *Hippopotamus* (ANU 075), Taolambiby, with chop-mark (c, d) but compare with chop-marked *Propithecus verreauxi* calcaneus (ANU 070) from Taolambiby (e, f).

At Ambolisatra (NISP = 498) the larger bones were mostly whole, in association, and extensively abraded. Some apparent cutmarks at low magnification on *Hippopotamus* bones were resolved into typical abrasion damage under SEM (Fig A in S1 File). No cutmarks were recorded. Megafaunal bones excavated in the Akororohe locality at Itampolo exhibited diverse skeletal elements as whole bones, hinting at associated deposition. They also showed some potential cutmarks that manifested as scoring and abrasion under SEM (Fig B in S1 File). One bone was cutmarked, a *Hippopotamus* jugal (Fig 3). The anatomical location of the mark is unusual for butchery, and as the specimen came from a modern well in which megafaunal fossils were dug out by local people it might have been damaged during that activity. It is dated 1595–1415 cal B.P. (Table G in S1 File). For Itampolo megafauna (NISP = 702) the cutmark rate is 0.14%.

**Fig 4:**
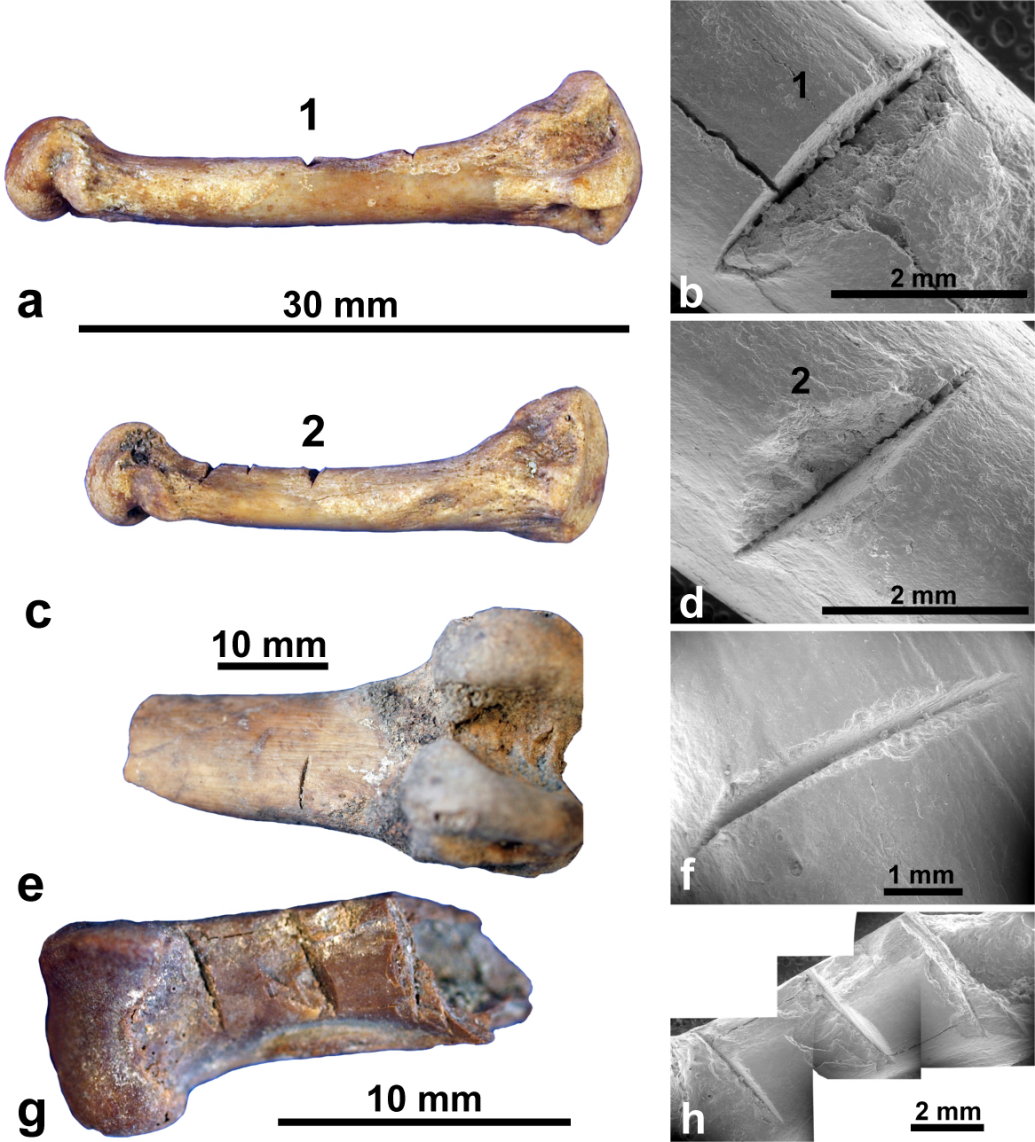
Cutmarks on extant taxa bones at Taolambiby: 4th (a, b) and 5th (c, d) metacarpals of *Cryptoprocta ferox* (ANU 007a and ANU 007b) in normal light and SEM; similarly cutmarked femur (ANU 130–2) of *Propithecus verreauxi* (e, f). *P*. *verreauxi* metapodial (ANU 043) with multiple cutmarks (g, h). See Fig E in S1 File for cross-section of cutmarks produced by MZ16 stereoscopic microscope.

At Area 1, Taolambiby, 32 cutmarked bones of Verreaux’s sifaka (*Propithecus verreauxi*) and five of the fossa (*Cryptoprocta ferox*), both extant taxa, were found in charcoal-enriched sediments lying immediately above yellow-grey compact sediments in which megafaunal bone occurred (Fig K in S1 File). Four samples indicate probable butchery of the extant taxa at 1150–950 y B.P. (c.f. ages of 1015 y B.P. to modern on 13 butchered *Propithecus* bones in the Walker collection; [43]). The sharp definition of the cutmarks (Fig 4 and Figs C-D in S1 File), and a typical cross-sectional profile of them (Fig Ee in S1 File) contrasts with marks on extinct lemur bone that appear superficially as cutmarks but are shown more probably as taphonomic damage under SEM (Fig 2 and Fig Ea-d in S1 File). For identified extant small mammals and birds (NISP = 415) in the Taolambiby assemblage, the cutmark rate is 8.91%. On the extinct megafauna at Taolambiby (NISP = 587), a possible chop mark was identified on a juvenile *Hippopotamus* metapodial dated 1260–1070 cal B.P. The example is atypical, including under SEM (Fig 3) because there seems to have been some bone growth around the cut. It might be from a natural injury, but if it is anthropogenic then the megafaunal cutmark rate at Taolambiby is 0.17%. The cutmark ratio between extinct megafauna and identified extant mammals and birds at Taolambiby is 1:52.

### Radiocarbon chronology of bone deposits

**Fig 5:**
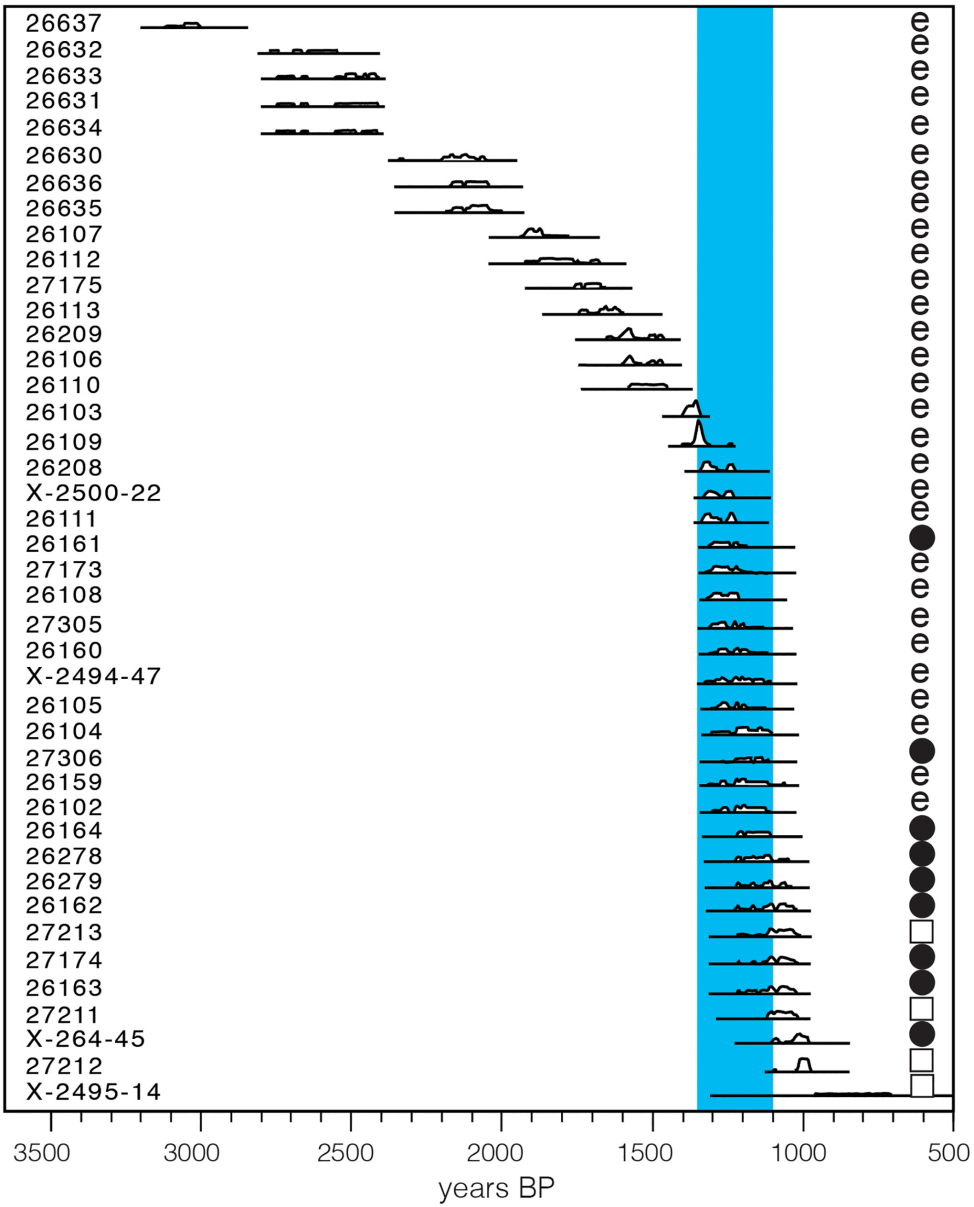
New AMS ^14^C dates for the three sites excavated in this research (see Table G in S1 File): e = bone of extinct fauna; filled circle = bone of extant fauna; square = charcoal sample.

Of 69 samples of bone (n = 65) and charcoal submitted to ORAU for AMS dating in 2011–2012, 27 bone samples failed due to low yield, all but one from extinct taxa. The failure rate suggests that some, possibly many, ^14^C dates on Madagascan megafaunal bone produced under earlier pre-treatments may not be reliable [29]. Our 42 results (Table G in S1 File) show that the latest megafaunal bone beds are relatively young (Fig 5). Ten bone samples date the bone bed at Itampolo, to 1832–1068 cal B.P., with four of them on *Hippopotamus* teeth at 1301–1068 cal B.P. Four samples date the Ambolisatra bone bed to 1315–982 cal B.P. The bone bed appears the same as that encountered in earlier excavations where it spanned 4965–2915 y B.P. on bone dates [17]. In the Area 1 excavation at Taolambiby, six megafaunal bone samples dated 1265–983 cal B.P. (excluding OxA-27175), while nine bones from extant fauna dated 1177–808 cal B.P. Four charcoal samples spanned 1057–554 cal B.P. Eight *Palaeopropithecus* bones from the Methuen collection, Taolambiby, were ^14^C dated to 3057–1918 cal B.P. encompassing the earlier ^14^C result [14] and confirming an older deposit in the site.

### Sedimentary record

Analysis of the Ambolisatra core shows that organic material began accumulating >7000 cal B.P., with a hiatus ~4500–1800 cal B.P. (Fig I in S1 File). Humid/mesic woodland with low levels of burning ~7000–4500 cal B.P. was replaced by xerophytic bush-savanna by 1800 cal B.P., the shift likely reflecting climate change and sea-level dynamics. Increasing burning 1600–700 cal B.P. probably reflects early human activity, at least by ~1000 cal B.P when there is a major increase in the incidence of macro-charcoal particles (Fig I in S1 File).

## Discussion

This research shows that large, newly-excavated, assemblages of megafaunal bone have almost no evidence of typical cutmarks. Nearly all the megafaunal bone modification appears taphonomic, including biting and gnawing marks by predators or scavengers (Fig F in S1 File), root etching, and several examples of chop marks incurred during excavation (Fig G in S1 File), as in other collections from Madagascar [14]. Much of the bone is broken and abrasion is ubiquitous. The subfossil bone beds accumulated in lakeshore sediments of grit, sand and silt, and at Taolambiby in particular there is also abundant tabular quartz sandstone from the adjacent cliffs. The movement of bone against stone could have involved downslope or water movement, but it is also consistent with trampling. *Hippopotamus*, crocodiles, giant lemurs and giant tortoises prior to the arrival of people, then people and livestock crossing the site daily to an adjacent waterhole, are the likely agents.

Strongly contrasting rates of bone modification are evident in our results. For the entire assemblage of megafaunal remains the cutmark rate is low (0.11%), as noted informally of northern Madagascan sites [6]. If perimortem activity is represented then it was not systematic butchery, as exhibited globally by abundant cutmarking, fracturing, burning and association with butchery tools in Holocene big-game processing sites. Flaked stone tools, exceedingly scarce in Madagascan archaeology [19], have not been reported from megafaunal bone sites. If iron tools were in use after about 1300 y B.P, as suggested in the damage on extant taxa bones at Taolambiby, then more substantial evidence of cutmarks could have been expected on the bones of extinct taxa. Instead, the frequency of cutmarks on megafaunal bone is the same, 0.10%, as that incurred accidentally in archaeological bone recovery.

Conversely, rates of typical cutmarks are much higher on bones of extant taxa, 9% overall in our material (14% for *Propithecus* and 50% for *Cryptoprocta*) and 29% on *Propithecus* bone in the Walker collection [14], suggesting that bone modification increased considerably after about 1200 y B.P. Butchery practice might have changed; small animals are routinely chopped into pieces in rural Madagascar [44], perhaps increasing the cutmark incidence. It is also possible that the advent of cutmarking on bones of extant taxa actually represents the beginning of all butchery in these sites, i.e. the age of human arrival. Cutmark rates are also relatively high, 6–9%, in some museum collections of megafauna (S1 File and Fig H in S1 File) and these can be hypothesized as reflecting perimortem activity <1100 y B.P., in ways that left more cutmarks than earlier, damage during bone recovery and handling, or acquisition from local people of bones that had sustained post-recovery damage. As no megafauna are dated younger than ~1100 y B.P. in our assemblages, the latter possibilities seem more plausible, but in any event the argument for IHCE 2000–2500 y B.P. on the basis of megafaunal cutmarks is clearly tenuous.

Recent palynological evidence of vegetation change suggests that anthropogenic impacts were later than argued previously. Forest burning >2000 cal B.P. is largely confined to the arid southwest, where natural firing is expected. Wetter regions show rises in charcoal abundance, around 1150–950 cal B.P. [16–18, 45, 46]. Intense local burning ~1000–400 cal B.P. coincides with climatic desiccation [45] and a shift from C3 herbaceous marshland to C4 dominated grasslands (Table F in S1 File), suggesting intensified human activity, including cattle herding and pastorally induced deforestation [28]. A stratigraphic record from Taolambiby, Area 3 (Fig J in S1 File), also shows a continuous rise in the abundance of charcoal pieces >125 microns beginning <1000 cal B.P.

Turning to megafaunal extinction, our data from the semi-arid southwest region refer to sites around small lakes that probably concentrated the distribution of some taxa, e.g. hippopotamus and crocodile, during terminal phases of bone accumulation; ~1350–1000 cal B.P. at Ambolisatra and Taolambiby and ~1900–1100 cal B.P. at Itampolo (Fig 5). Yet cutmarking is very scarce or absent on megafaunal bone in those sites and when it appears around 1200 y ago it is confined to bone from small extant taxa. This observation, together with the virtual absence of megafaunal bones in southwestern settlement sites [29]; and in middens dating 1400–1000 y B.P. [47], suggests that the role of human predation in regional extirpation of megafauna did not exceed “imperceptible overkill” [48], reflecting the vulnerability of generally conservative (K-selected), megafaunal life histories to modest increases in death rates by continual low-level hunting that left few traces in the sedimentary record. The youngest megafaunal dates ~1350–1000 cal B.P. at all three sites, in our data (Table G in S1 File), are consistent with the youngest ages on four genera of extinct lemur, 1460–1010 y B.P. [28], and semi-quantitative evidence of plummeting population decline in large megafauna around 1000 y B.P. [26] in the southwestern region. Megafauna might have disappeared earlier around focal water sources than regionally as some taxa, including elephant birds, survived up to about 600–500 y B.P. [28, 29], but there was, at least, a marked megafaunal decline around a millennium ago. As a whole, these palaeoecological data indicate no support for human activity in southwest Madagascar before 1500 cal B.P., but exhibit diverse evidence of human activity from about 1350 cal B.P. In addition to claims of earlier megafaunal butchery, however, it has been argued that colonization up to 5000 y B.P. is indicated by the chronology of Lakaton’i Anja archaeological site.

### Chronology at Lakaton’i Anja

At this rockshelter site (Fig 1) five layers contained chert tools, bone, shell and charcoal [19]. Layers 1–3 were dated stylistically on pottery and glass to <1450 y B.P., and to 1330–930 y B.P. by OSL. Layers 4–5 were ^14^C dated on charcoal to 1460–930 y B.P., but by OSL to 2700–2200 y B.P. (layer 4), and 4380–3470 y B.P. (layer 5). Explanation of the conflicting chronologies relied upon a differential displacement hypothesis in which the main OSL signals from “host sediments” (representing the original undisturbed deposits) also dated the archaeological remains in layers 4–5, except for charcoal selectively introduced from above by termites as a source of moisture [19]. However, large termite burrows from above had penetrated layers 4–5 and caused “significant [sedimentary] contamination” [19]. In five of the six OSL samples [19], 28–40% of quartz grains belonged to introduced populations. The displaced sediments were generally younger, indicating downward displacement; e.g. the 32.5% of minor population grains in OSL sample ANJA K3/A dated ~600 y B.P. compared to 1330 y B.P. for the host sediment, and most minor grain values in other OSL samples suggest ages of ~1000 y B.P. or younger. Pottery and chlorite schist sherds dating <1000 y B.P. were displaced downward. A glass bead dating <1250 y B.P. from layer 1 was recovered in layer 5. As the bone was from small animals, and pieces of flaked stone were less than 2 cm in length [19], all of the cultural material was susceptible to displacement through bioturbation, not just charcoal (S1 File).

If the site had been occupied periodically for >3000 y some variations in material sources or technical traditions might have been expected, but even minor materials such as red chert and quartz crystal occurred in upper and lower layers and the same flake tool industry throughout Lakaton’i Anja strata as at Ambohiposa, the only other site with flaked stone tools in Madagascar, where the lowest layers date <1100 y B.P. [19]. The OSL ages on host sediments, and displacement of charcoal samples from higher layers, are probably correct, but it is most unlikely that midden and artefacts were not also displaced in the extensive bioturbation that moved upper sediment to lower levels. A simpler explanation of the Lakaton’i Anja evidence, therefore, is that all of the scarce archaeological material in layers 4–5 came from rich deposits in upper layers by the extensive bioturbation recorded in the stratigraphy and OSL samples, in which case ^14^C ages directly upon archaeological material in layers 4 and 5 suggest site occupation began <1500 y B.P.

## Conclusions

1. Microscopic examination of freshly-excavated bone, in very much larger assemblages than have been analysed previously, revealed cutmarks on 9% of samples from extant taxa at Taolambiby, dating 1236–927 cal B.P., but only 0.17% (one possible item), on megafaunal specimens of similar age, 1282–1062 cal B.P. At Itampolo the megafaunal cutmark rate was 0.14% (one item of suspected post-mortem damage) and no cutmarks were observed on bones from Ambolisatra. No cutmarks were found on extinct lemur bone dating 3057–1918 cal B.P. from the Methuen Taolambiby collection. The null hypothesis that bone damage in Madagascan material is taphonomic in origin cannot be rejected for specimens dating earlier than about 1300 y B.P.

2. At Lakaton’i Anja extensive bioturbation, signalled stratigraphically and by scattered OSL datapoints, indicates downward displacement of cultural material with associated sediment to form ~30% of sediment in lower layers. Estimated OSL ages on that sediment are more consistent with <1700 y B.P. ^14^C and typological ages on the cultural material than with OSL ages of 4400–2200 y B.P. on host sediments. The Lakaton’i Anja chronology, which is the keystone of mid-Holocene colonisation hypotheses, therefore needs further testing. If bone, marine shell and charcoal samples from layers 4–5 produce ages similar to OSL results on host sediments, then deposition by contemporaneous habitation is indicated; if the ages remain consistent with those known already then wholesale sample displacement is the more probable explanation.

3. Anthropogenic vegetation changes began 1600–1000 cal B.P. and megafaunal extirpation ~1200 cal B.P. in the sites we investigated. Similar research is required elsewhere in Madagascar to assess the generality of our results, but they bear consideration now in thinking about decline and extinction of megafauna [26, 49], a proposed early hunter-gatherer phase, putatively African, prior to Austronesian arrival [21–24], and transoceanic voyaging from Southeast Asia >1500 y B.P. [1, 2, 20, 21]. On evidence here, human occupation in Madagascar cannot be inferred convincingly before ~1350 cal B.P. and an additional range of evidence converges on 1350–1100 y B.P. for initial colonization: genomic and linguistic data indicating migration ~1350–1200 y B.P., archaeological evidence of settlement sites 1300–1100 y B.P. [22, 25, 29]; the earliest pottery of *Arca* shell-impressed wares dating ~1200 y. B.P. in the Comoros, and Triangular Incised Ware dating 1350–950 y B.P. in East Africa [29]; cattle arriving ~1200 y B.P. [50] and Asian crops getting to the Comoros and Madagascar, 1200–950 y B.P. [25]. The initial colonization of Madagascar may have involved planned migration by maritime trader-farmers, as occurred in the late Holocene South Pacific and North Atlantic islands.

## Supporting information

S1 File Research on sources of Madagascan IHCE estimates. (PDF)
