# Supplementary material for: New evidence of megafaunal bone damage indicates late colonization of Madagascar
Source: PLoS One. 2018 Oct 10;13(10):e0204368. doi: 10.1371/journal.pone.0204368 (PMC6179221; doi:10.1371/journal.pone.0204368)
Supplement: S1 File — (PDF) [file pone.0204368.s001.pdf]

S1 File. Research on Sources of Madagascan IHCE Estimates

Variation in IHCE. In Text Fig 1 there are 51 IHCE (Initial Human Colonisation Estimates), illustrating the changes that have occurred since 1990 in thinking about when Madagascar was first colonised. Cited references #5-50 in Text Fig 1 are in the main text; references #51-73 are in the S1 File references, listed below. The sample includes all the references we could find that discuss colonisation age rather than simply cite earlier sources. It therefore represents the approximate distribution in the literature from 1991 to 2017 of different evidential sources, methods and conclusions about colonisation age. Evidential sources shown are not always the same as methodological approaches. In genomic history, for example, colonisation ages can be measured by genetic methods [8] or assumed on the basis or archaeological, linguistic and paleoenvironmental data [70]. Some papers appeal to more than one evidential source and we show what appears to have been to be the predominant source used.

Text Fig 1 illustrates the main trends in estimating Madagascan colonisation age. Before 1990, historical linguistics and archival history - notably Greek and Roman records from around 2000 y ago that were argued to implicate Madagascar - together with results from early academic archaeological fieldwork [3, 4, 6], suggested that people arrived first in Madagascar in the approximate period 2000-1350 y B.P. The early end of that range was then supported, initially in 1991 [12], by radiocarbon-dated megafaunal bones exhibiting evidence of cultural modification. An hypothesis of butchered megafaunal bone, to some extent buttressed by evidence of environmental change [14, 18] and extending to about 2500 y B.P., became subsequently the mainstay of IHCE until 2011, when even older instances of assumed megafaunal cutmarks, dating to about 4200 y B.P., were soon matched by OSL dates at Lakaton'i Anja. That sedimentological evidence then became the basis of estimating Madagascan colonisation as occurring up to 5000 y B.P. [22-24]. We provide here a fuller account and critique of research on modified megafaunal bones and of the Lakaton'i Anja chronology.

Critique of earlier research on modified megafaunal bone. There have been three particular studies of bone damage.

(I) On seven *Hippopotamus* femora dated >2000-1600 y B.P., from Ambolisatra/Andolonomby and Lamboharana in the Grandidier collection (Muséum national d'Histoire naturelle (MNHN) Paris) convincingly anthropogenic chop marks were taken as perimortem because they were reproduced experimentally on fresh cow bone and not on subfossil *Hippopotamus* bone [12]. Field experience, however, shows that subfossil bones embedded in stiff sediment exhibit clean chop marks when struck accidently by metal blades (below). A *Hippopotamus* femur from Ambolisatra (femur 4) has a series of long, planar chop marks, most at around the mid-shaft, which appear to be preparation either for snapping the bone in two or for sharpening the broken end. Similar working occurs around broken shafts in three additional hippopotamus femora from Lamboharana, a site that no longer exists. The marks are cultural but they are not characteristic of butchery damage and they could have been made at any stage in the history of the bones. Subfossil bone has sometimes been used as raw material for artefacts, and re-shaping of hippopotamus femora and drilled *Daubentonia robusta* incisors from Lamboharana, are perhaps evidence of it [14]. No sawing, drilling or

shaping of Madagascan megafaunal bone is reported in material freshly excavated since 1990, at least.

(II) A later study of Madagascan bone damage [14], inspected 297 lemur bones from Taolambiby and Tsirave; 265 were from extant taxa, nearly all *Propithecus verreauxi*, and 29% of those exhibited cutmarks, of which those figured are convincingly cultural [14: Figures 6, 7]. Of 28 extinct lemur bones in the Methuen collection (Oxford University Museum of Natural History (OUM) Oxford), 10 had bone damage (53 apparent cutmarks) attributed to butchery [14: 729]: One bone, from Taolambiby, was dated 2366-2315 y B.P. In defining criteria for identifying butchering marks, it was argued [14] that breakage, spiral fractures, splintering, percussion striae, scratches and abrasion can be of non-cultural origin and therefore not definitive in judging the likelihood of cultural agency. In addition, the common criterion of narrow V cross-sections for cut-marks was rejected on the ground that both lithic and metal blades could produce V or U-shaped kerf floors depended on the way in which they were used. The marks were generally very small in relation to the bones, averaging 5.6 mm in length, and their depth and morphology were difficult to define because the bones were coated in incompletely removed curatorial wax. No cross-sectional images of marks on bones from extinct species were shown. The method for identifying cut-marks [14] was largely restricted, therefore, to patterning or redundancy in marking and assumed signs of purposefulness in the location of tool marks. Interpretation of the marks as butchery evidence relied particularly upon their distribution to distal (41%), and proximal (36%) bone regions, as opposed to the midshaft (23%); but as epiphyseal surface areas of humeri and tibiae, the most common bones in the sample, are ~1.5 times the area of the mid-shaft, significant locational variation in damage frequency is doubtful.

In addition, the criteria [14] fail to take into account that bones of a similar shape are likely to move, and be damaged taphonomically, within sediment in similar ways, producing patterns of damage distribution and extensive surficial damage that include locations common to butchery. Perez et al. [14: 729] observed that, "bones in our sample exhibited very few nonhuman taphonomic changes", but they did not test that matter explicitly and the role of trampling was not explored. This is a significant methodological lacuna because post-mortem trampling of bone can produce surficial damage that is not only similar to that from butchery but also distributed in a similar way. Experimental research has shown that anatomical location is not a useful attribute in differentiating cut marks from trampling marks [47]. The Perez et al. [14: 738] claim to discovery of "the first definitive evidence of butchery of giant lemurs" is therefore open to question.

III Numerous fossil bones of Madagascan megafauna have been collected in the extensive cave system at Anjohibe, NW Madagascar, especially in areas where there are pitfall openings in the cave ceilings, and an underground river may also have been an agency of deposition. Burney et al. [31: 762] excavated a dense deposit of extinct *Hippopotamus* bone, finding "no evidence to implicate humans in the animal's death". They obtained a collagen date of 3730 ± 70 yr B.P. on one bone at that site, and concluded that the animals had died long before the arrival of people. From the same cave chamber (Salle R. de Joly), Gommery et al. [15: 2] recovered three *Hippopotamus* bones that "show a significant quantity of marks due most likely to human activity". Two are dated indirectly to ~3800 and ~3300 y B.P. The bones have 34 damage marks which were taken as evidence of butchery. Images of the bone modification show scoring marks, 3-11 mm long, in patches. Most of the marks have broad, shallow floors. Some are deep and narrow but they appear to lack the cleanly sliced edges typical of cutmarks. In the images [15] they have features suggestive of scavenger chewing and biting. One bone has a possible chop

mark, but that would not be unexpected in a site that has been targeted by fossil hunters since the 1930s. On present evidence, the modified *Hippopotamus* bones recovered from Anjohibe do not appear to have been butchered.

New analysis of older collections. We re-examined the Methuen collection of extinct lemur bone used by Perez et al. [14], with reference to criteria for distinguishing cutmarks from damage by trampling [38]. Initial examination was under X10 light magnification but after using acetone to remove nearly all of the wax coating in areas of interest on these bones the damage features were imaged under higher magnification. The material we inspected, and the distribution of different kinds of observed surficial bone damage, is noted in Table A. We reached different conclusions from Perez et al. [14] about the cause of this damage, and they are best explained with reference to specimen OUM 14342A (Text Fig 2), a *Palaeopropithecus ingens* left humerus [13, 14]. Of the 45 cutmarks identified on the eight *Palaeopropithecus* bones thought to exhibit evidence of butchery, 40% are on this one specimen [14: 730, Table 2], and it exhibits the greatest variety of damage morphologies. The main area of damage consists of nine "multiple cutmarks" [14: 738] near the distal end. These lie within a patch (35 mm X 7 mm) of heavy abrasion and pitting, suggesting that scoring and abrasion formed part of a single damage event. This is also implied by the changing orientation distally of the grooves from near-perpendicular to increasingly oblique along the ridge on which they lie**.** There is no specific reason to think this trend would result from an episode of butchery, and there is a plausible taphonomic explanation. If the bone was rotated under pressure about the longitudinal axis of the shaft then scoring agents would create perpendicular marks along the mid-shaft but marks that were progressively more oblique along the outward curving ridge, the pattern that is, in fact, observed. Comparison of attributes of the damage with criteria in Dominguez-Rodrigo et al. [42: 2466-2467] showed that the damage was most probably from trampling, particularly on grounds of mark trajectory, groove cross-section and overlapping striae (Table B).

There is some overlap in criteria between trampling and the use of retouched stone edges, notably in the cross-sectional shape of grooves, but overlapping striae, low shoulder effect and curved groove trajectories also favour trampling (as does the absence of any flaked stone tools at Taolambiby). Dominguez-Rodrigo et al. [42] argue that unretouched flakes (or metal edges) produce narrow V cross-sections to grooves, straight scores and virtual absence of overlapping striae. These score low in the present case. Additional 'cutmarks' on the midshaft and proximal areas of OUM 14342A consist mainly of faint scratches or, in one case, a shallow score with a broad (3.5 mm wide) floor. That mark, and two wide, shallow, intersecting marks set in a broad impact scar on the mid-shaft are possibly evidence of spade contact during bone recovery [cf. 14: 732]. None are identifiable as characteristic butchery marks (as identified conventionally, e.g. 33-39, although what is demonstrably characteristic is currently debated, 40-42).

We conclude that, under SEM and microscopic cross-sectional profiling (Text Fig 2), the 18 cutmarks identified on *Palaeopropithecus* specimen OUM14342A, have relatively shallow, broad and blunted features more typical of bone abrasion generally, and perhaps especially of bone trampling,.

In addition to damage already mentioned, there are numerous circular dents along the shaft of OUM 14342A, five up to 2.5 mm in diameter, and one of 5 mm diameter with a 50 mm crack originating from it. These might be tooth damage from scavengers, such as crocodile, or pressure pits caused by trampling bone against pieces of stone. Examination of other bones in the Methuen collection from Taolambiby cast doubt on the "chop mark"

on OUM 14342B [14: 732]. The mark has irregular and rough breakage surfaces suggesting that a piece has been broken out under pressure or knocked out by a blunt object. The four grooves on OUM 14346aA do not have characteristics of cutmarks and they are associated with numerous fine abrasions in the same direction, suggestive of an episode of abrasion. On OUM 14346aC, D, G and L, the damage consists only of faint scratches and associated abrasions, although G has three dents. Similar damage is seen on various other *Palaeopropithecus* bones from Taolambiby which were not regarded as cut-marked by Perez et al. [14]: e.g. OUM 14346a F, J, K, P. Sample OUM 14346a E is a radius in which the distal shaft appears to have been chewed. Overall, the marks on all of the bones we examined from the Methuen collection differ markedly from the clearly cut-marked *Propithecus* bones we collected from Taolambiby in that they lack clearly defined edges, they often curve, they vary significantly in orientation and depth, there are no chatter marks present and cross-sectional profiles are much more consistent with abrasion than butchery. A convincingly chopped but undated *Pachylemur* femur from Tsirave [14], is an exception, and 13 additional *Pachylemur* femora from Tsirave, all undated, are stated as cutmarked, without details. A human modified *Aepyornis* tibia dated to 1880 ± 70 yr B.P. is reported from Itampolo but no details have been published [55].

In the Grandidier collection, MNHN, 325 *Hippopotamus* long bones and 124 of extinct lemur were inspected under X10 hand lens. Cutmark rates of 9.2% for the former and 6.4% for the latter were observed; c.f. a cutmark rate of 6.4% on *Pachylemur* femora in Madagascan collections [44]. Much of the damage in the Grandidier collection is by chopping. Thick-walled shafts of *Hippopotamus* femora, in particular, seem to have been chopped out for use as artefactual raw material. When this occurred is unknown. The bones from Lamboharana and Ambolisatra were collected 1898-1901 by Grandidier or collectors employed by him [17] and some might have been recovered from local people rather than directly from subfossil sites. Details follow:

Archaeolemur: small slice/score marks 3-5 mm long on: MAD7680 (Tib); 7671 and 8888 (Fem). *Palaeopropithecus* humeri: MAD8797 5 mm slice/score; 1584 broken cleanly across; 8598 chopped; 8604 sawn in half. Megaladapidae: MAD1564 (Fem) sawn through. *Hippopotamus*: MAD7903 (Rad/Ul) 35 mm oblique slice/score; MAD7894 and 8509 (Tib) both with 6-7 mm tooth punctures, and latter has five broad scratches. On femora: Substantial chopping (5 to 16 blows) with metal implement around circumference of bone with subsequent snapping to separate epiphysis from shaft on MAD1708, 1709, 1710, 1711 (these are the samples specified and dated in reference 12); similar chopping on MAD8392 and 8398; mid-shaft chop marks on MAD8390, 8394, 8396, 8474, 8493, and 8497. Femur MAD8393 has grooves in shaft cut by a grinding wheel. There are small scores or cutmarks on the shafts of MAD422, 8397, 8485, 8719 and what seems like bite damage on MAD8477 and 8716. Two femora in bone series MNHN2007-1 have single scores 6 and 16 mm, and a third has an 11 mm long flake scar, all on the shafts. Therefore, of 23 femora with damage, one has been cut by a grinder, four have bite marks, six have small scores or cutmarks, and 12 have been chopped by a metal implement, in some cases clearly for purposes other than butchery. On humeri, MAD8356, 8728 and one in series MNHN2007-1 there are dents or punctures that appear to be tooth marks. MAD8375 has a diagonal score or cutmark and a juvenile humerus, MAD7899, has four small scratches up to 2 mm deep.

In summary, the incidence of damage on bones of extinct megafauna in the Grandidier sample is about 10%, but as the collecting strategy is unknown, it is impossible to tell whether this is representative of bone damage rates in the original deposits. Damage is less (3%) amongst lemur bone than *Hippopotamus* bone (9%), and in both

cases half the damage seems to have an industrial origin, either in chopping hippo femora to remove the heavy, cylindrical shafts for some purpose, or in sawing the lemur bone (Table C). The intent of this damage remains uncertain, and implements fashioned from the long bones of *Hippopotamus* or giant lemur do not seem to have been recorded from archaeological sites.

New bone recovery at subfossil sites in Southwest Madagascar. *Ambolisatra.* In addition to test pits around the swamp at Ambolisatra/Andolonomby (here, as elsewhere, many localities have multiple names) we made a 4 m^2 excavation 10 m NW of the 9 m^2 pit excavated by Burney [51], Larger bones were extracted by hand from the sticky clay, but retrieval of smaller bones required the sediment to be dried before water sieving. Although numerous bones were found in the main pit, starting at a depth of 130 cm and with a particular concentration from 155-170 cm, relatively few individuals are represented (Table D). The clay content increased down to a hardpan at approximately 180 cm depth; bone abundance decreasing with depth from 165 cm down to the hardpan. No bone was found below the hardpan and the pit was terminated at a depth of 210 cm. Burney [51] reported bones of extinct megafauna between 180 and 260 cm before meeting the hardpan, thus the bone-bearing unit appears thinner in our excavation, possibly because our work was located further back from the centre of the swamp. The bulk of the specimens represented associated *Hippopotamus* material and the majority appears to have been from a single adult and a single juvenile. Diversity was low (Table D), similar to that found by Burney [51], although several bones belonging to a large extinct lemur were found, a taxon not reported by him. There were no indications in terms of bone distribution that the site represented a butchery site or any other kind of anthropogenic accumulation; the most probable scenario being that the bone accumulations are natural, autochthonous death assemblages. The bones from Ambolisatra excavated by us do not show any signs of anthropogenic influence in terms of surface modification. All marks on bone surfaces could clearly be attributed to abrasion damage (Fig A).

Itampolo. This site consists of a series of small, waterlogged, saline depressions, two of them named Ankororohe and Andronovony. We excavated four 1 m^2 pits at Ankororohe to a maximum depth of 140 cm in the vicinity of previous excavations [51]. The sediment was wet-sieved through 2 mm mesh sieves. Bones were found in multiple levels in these pits, from 50 to 140 cm deep. The majority of bones belonged to *Hippopotamus* sp. but it was unclear whether this material represented associated skeletons. We found that the bones from Ankororohe were extensively marked by abrasion (Fig B). The Andronovony location at Itampolo is a well shaft approximately 1 m in diameter dug in the latter part of the 20th century. It is still used as a well and subfossil bones are recovered from it by local people, including a complete *Hippopotamus* skull, subsequently painted red. We purchased it as a gift to the Musée d'Art et d'Archaéologie, Antananarivo. Forty-five *Hippopotamus* bones were collected, representing at least three individuals as well as the bones of several other large taxa. A fragment of the anterior zygomatic (jugal) of a *Hippopotamus* sp. from the Well Pit excavation shows a series of marks along the ventral border of the orbit that conform to the morphology expected for cut-marks (Text Fig 3). This bone was ^{14}C dated to 1409-1537 cal B.P. (OxA-26209, Table G), but considering its provenance the cut marks might be much younger than the bone.

Taolambiby. Despite various collections being made at this site no detailed stratigraphic reconstruction is available except for Raison and Vérin [74]. The Methuen

and Lamberton collections are both missing stratigraphic provenance and collection strategy data. Walker did not excavate at Taolambiby, but collected bones along the stream bank eroding out of a fossiliferous layer of black sand, 50 cm thick, and 60 cm below the modern soil, although occasional bones were found in a deeper layer of fawn sands [14]. We made three excavations at Taolambiby (Fig K) in an attempt to better understand the stratigraphy of the site and to ensure that fossil specimens were collected *in situ* where possible and that they did not represent recently reworked specimens or surface scatter. The stratigraphy of the Taolambiby bone deposit is similar across the three excavated areas, although differing in detail. The Area 2 and Area 3 excavations were in a part of the site where large sandstone outcrops have prevented the extent of erosion evident in Area 1, and the similar stratigraphy is illustrated here by Area 2 (Fig L). This shows a sandstone base, with several levels cut into it, presumably representing former lake levels. At the lowest level (Fig L, layer 5) there is pale grey, very compact, fine sand and clay with no bone apparent. This may have been part of the lake floor sediment. Above it (layer 4) is a 0.3-0.4 m thick deposit of yellow-grey fine to medium sand containing sandstone slabs and pieces, especially near the base. This is a bone layer, apparently on the edge of the lake, and it contained fragmented megafaunal bone. It is capped by an indurated and slightly cemented grey-brown sand, layer 3. The medium, compact, sand deposit above that, layer 1, is dark-grey and almost divided in two by a very compact silty lens (layer 2) containing numerous flecks of fine white sand and pieces of sandstone. Charcoal occurs sparsely above the lens. There was no bone in the dark-grey sand deposit apart from one fragment of extinct lemur bone found above the dividing lens. A plain pottery sherd was also found there. We interpret the stratigraphy to record the last phase of subfossil bone deposition on the former lake shore which was capped by successive sedimentary deposits in which, near the top, there is evidence of cultural activity. Only one megafaunal bone (*Hippopotamus* tooth) from Area 2 produced a radiocarbon age: OxA-27305, 1004-1182 cal B.P. (Table G).

At Area 1 the exposed surface is eroded and some material in the uppermost 5 cm, or in erosion runnels, has been redeposited through slope erosion, including bone and carapace from the extinct tortoise (*Geochelone*), which appears to have come from exposed remains near the base of the low cliffs immediately above the site. A goat tooth was recovered in the uppermost sediment but an attempt to date it by ^{14}C failed. The relatively undisturbed stratigraphy here consists of 5 cm of loose grey sand above a 10 cm brown, blocky, sand and humus soil horizon with humus and rootlets in which there is no bone. From 15 to 30 cm depth is a mid to pale brown, compact sand with a charcoal-enriched lens running through it at 25-27 cm deep, in several places suggesting small hearths. There is fragmented bone of extant taxa, one *P. verreauxi* metacarpal being partly burnt and others clearly cut-marked at this level. Below the charcoal band, at 30-35 cm deep there are pieces of megafaunal bone. Radiocarbon dates were obtained on the charcoal in, and immediately below, the charcoal lens, on the associated bone of extant fauna and on samples of extinct fauna from below the charcoal lens. A compact medium, yellow-grey sand, silt and clay with occasional pieces of megafaunal bone in it, extended to 60 cm depth. Throughout the stratigraphy were numerous pieces of sandstone from boulder to pebble-sized.

New bone analyses. The megafaunal taxa expected in Southwest Madagascar, and those found in our excavations are shown in Table E and a full list of recovered fauna in Table D. All bones that exhibited cuts, chops, slices or anything else that could be taken for cutmarks were examined and imaged. The marks were cleaned with acetone, which made

their overall shape much clearer. In nearly all cases, the marks had a shallow, open U-shaped cross-section, often with rough or pitted walls. This contrasts with the V-shaped, sharply defined cut-marks seen on *Propithecus* bones from Taolambiby. Yet, the typology of damage, even if it is accepted as a valid criterion for distinguishing cultural from taphonomic modification, is not the only consideration here.

Cultural modification is demonstrable on a small proportion of bones from extinct megafauna in museum collections, but it is impossible to show that it was perimortem or to rule out modern damage. In our excavations we noticed that occasional inadvertent damage by fieldworkers using the sharp, crescentic-edged Mahafaly spades to clean excavation baulks produced planar cuts on subfossil bone shafts and clean chops across epiphyses, if the bone was still bedded tightly in damp, dense silt. The damage is identical to that on some examples of megafaunal bones in museum collections. Conversely, the absence of convincingly large and numerous cutmarks on megafaunal bones from newly-excavated sites raises a question about the perimortem status of the damage in the museum material. All that is certain here is that the radiocarbon age of bones alone is an unreliable guide to the age of the cultural damage they might have sustained. Potentially reliable dates are restricted to bones recovered from recorded stratigraphic provenances in controlled excavations, where the existence of prior damage of plausibly cultural origin can be verified. Our few examples do not quite meet that standard. The marks on the *Hippopotamus* jugal at Itampolo are convincingly cultural but the association of recovery from a modern well raises a question over the source and age of the damage. At Taolambiby, there is a good provenance for a possibly cutmarked *Hippopotamus* metapodial, but the cultural origin of the damage is not entirely convincing.

In summary, we agree that modification of *Daubentonia* incisors and an *Aepyornis* tibiotarsus, are cultural, but not from butchery [14: 723] and we add damage to the hippopotamus bones from Ambolisatra and Lamboharana to that conclusion. Our re-assessment of the damage on *Palaeopropithecus* bones from the Methuen collection, contradicts the hypothesis that scoring marks on the bones are from butchery and suggests by the use of modern criteria for bone damage morphologies that most of it is more plausibly ascribed to taphonomic factors. Some damage, such as chop marks, may have been caused by digging bones out of the stiff, damp silt sediments at Taolambiby. Consistently with the thrust of this argument, our review of surficial damage on 2756 bones and bone fragments from our excavations indicates that butchery can be seen clearly on remains from extant taxa, but it is vanishingly scarce on megafaunal bones. To assess the significance of this conclusion it is necessary to consider the pattern of radiocarbon dating on extinct megafaunal remains.

Radiocarbon determinations. All samples were dated at the Oxford Radiocarbon Accelerator Unit (ORAU) at the University of Oxford. Pretreatment methods for bone and charcoal samples are described *inter alia* by Brock et al. [75]. The bone pre-treatment procedure involved a simple ABA treatment followed by gelatinization and ultrafiltration. Samples were treated with 0.5M hydrochloric acid (3 or 4 rinses over ~18 hr), 0.1M sodium hydroxide (30 min), and 0.5M hydrochloric acid (1 hr) with thorough rinsing with ultrapure water between each reagent. The crude collagen was gelatinized in pH 3 solution at 75 C for 20 hr and the resultant gelatin solution filtered using a cleaned Ezee-filter. The filtrate was transferred into a pre- cleaned ultrafilter (VivaspinTM 15–30 kD MWCO) and centrifuged until 0.5–1.0 mL of the >30 kD gelatin fraction remains. The gelatin was removed from the ultrafilter with ultrapure water before being freeze-dried.

Charcoal samples underwent ABA pre-treatment consisting of an initial

hydrochloric acid wash for ~20 min or until effervescence had finished, a sodium hydroxide base wash for 20 min, and a final acid wash for ~1 hr. Charcoal samples were not identified to wood taxa. They, and earlier charcoal samples from megafaunal bone sites and sedimentary cores, must be regarded as maximum ages, given the possibility that the charcoals incorporate "old wood", a significant problem in constructing IHCE on oceanic islands with long-lived trees.

The dates are uncalibrated in radiocarbon years B.P. (Before Present-AD 1950) using the half-life of 5568 years. Isotopic fractionation was corrected for using the measured ^{13}C values on the AMS. The quoted ^{13}C values were measured independently on a stable isotope mass spectrometer (to ±0.3 per mil relative to VPDB). Dates were calibrated using CALIB 7.1.0 using the SH Calibration curve [76] with date ranges at two sigma (95.4%). The results are in Table G.

Comments on results: OxA-X-2500-22. Bone: *Pachylemur* cf. *insignis* (CRA=1283 ± 24, ^{13}C=-21.79). This sample gave a low pretreatment yield (1160 mg gave 3.01 mg collagen, which is 0.3% wt. collagen), which was less than the acceptable threshold of 5 mg and 1%wt. As a result there is a slight health warning on this determination.

OxA-X-2464-45. Bone: cf. *Eulemur* (CRA=1064 ± 30, ^{13}C=-20.06). This sample was small, only 180mg so around 20% of required. In addition, the yield of collagen extracted was low (3.2 mg). This was less than the minimum threshold but all other measurement parameters we check were acceptable. The sample has a slight health warning on this determination.

OxA-X-2494-47. Bone: Hippo sp. (CRA=1231 ± 27, ^{13}C=-16.14). This sample gave a low yield of collagen (3.76 mg) which is less than the 5 mg minimum and produced less than 1% weight collagen. As a result there is a slight health warning on this determination.

OxA-X-2495-14. Charcoal (CRA=810 ± 100, ^{13}C=-26.68). The errors on this wheel were higher than normal because of scatter in the standards.

Age ranges in sites investigated. Radiocarbon dating has changed considerably over the period in which it has been applied to bone from Madagascan sites; from conventional to AMS methods and especially in pre-treatment protocols. All four of the bones we dated from Ambolisatra (Table G) were in the range 982-1315 cal B.P. which fits at the younger end of the age range, ~1070-1980 cal B.P., of the majority of bones ^{14}C dated from the site by Crowley [26]. Of her 38 dates, four fell in the range 2160-2850 cal B.P. and one 5770-6100 cal B.P but the stratigraphic provenance of all specimens dated in that study was unknown. MacPhee and Burney [12] published a date of 1720-1980 y B.P. for a *Hippopotamus* femur from Ambolisatra with purported cut-marks. The disparity in dates between those produced in our study and the older dates in MacPhee and Burney [12] and Crowley [26] probably represents sampling of chronologically more diverse deposits in earlier research, and perhaps differences in the precision of ^{14}C bone dating techniques. The Burney [51] excavation was about 10 m further into the swamp than ours, and it has deeper and thicker deposits possibly representing a longer period of deposition. Of the ten bones ^{14}C dated in our study from Itampolo, bones from Ankororohe ranged in age from 1068-1832 cal B.P. and those from Andronovony ranged from 1384-1823 cal B.P. (Table G).

All of our excavated and successfully dated bones from Taolambiby were in the range 808-1702 cal B.P. while the re-dated extinct lemur bones from the Methuen

collection at Taolambiby ranged from 1918-3057 cal B.P. (Table G). This may indicate that our excavations did not sample the layer from which Methuen collected extinct lemur bones with purported cut marks. However, no bones from the stratigraphically deepest of the excavations (Area 2, Fig L) yielded dates. This was due to insufficient collagen preservation, so the age of fossil material from Area 3 (which included *Hippopotamus* and extinct lemur bones) remains unknown. All bones from the white sandy unit at the base of our excavations also failed to yield dates but, given their stratigraphic position, they are likely to be older than the dated bones we collected and hence of a similar age to the bones from Methuen's collection. The *Hippopotamus* metapodial dated 990-1181 cal y B.P (OxA-X-2494-47). is important because it is the only example of a possibly cutmarked megafaunal bone that we found at Taolambiby and it is in the same age range as the cut-marked *Propithecus* bones at the site (Table G).

Reconsideration of the Lakaton'i Anja chronology. Two cultural layers were recognized during excavations to about 0.4 m deep in the Lakaton'i Anja rockshelter in 1986 [5]. Both contained local pottery, with imported ceramics confined to the upper layer. This stratigraphy corresponds to layers 1-3 and upper layer 4, as defined in the 2011 excavations [19]. There are no radiocarbon dates for layers 1 and 2, and ages of 1300 ± 80 y B.P. (Beta 18424) and 1680 ± 65 y B.P. (Beta 29946) by Dewar and Wright [6: 429], on charcoal samples refer to layers 3 or 4. Four additional ages, given without further details, were: A.D. 960, 968 and 999 from upper, and A.D. 405 from lower, levels of the lower cultural layer in 1986 [5].

In 2011, two 1 m^2 units (J, K) were excavated in sediments of silty sand at Lakaton'i Anja [19]. They contained cultural material to about 0.7 m deep in which 5 layers were recognized. Chert flake tools, bone, shell and charcoal occurred in all layers, while local and imported pottery and glass beads dating stylistically to <1450 y B.P., were almost entirely restricted to the upper layers 1-3. Three AMS radiocarbon dates were obtained on charcoal samples: 1460 ± 40 y B.P. from layer 4A; and from layer 5A, 1070 ± 40 y B.P. and 930 ± 30 y B.P. They are not in expected age order. Six samples of quartz grains were analysed by the single grain OSL technique. In unit J, samples from levels 2/3A, 4A/B and 5 B/C gave ages of: 0.93 ± 0.09, 2.70 ± 0.23 and 4.38 ± 0.40 ka respectively. In unit K, levels 3A, 4A and 5A/B gave ages of: 1.33 ± 0.13, 2.21 ± 0.19 and 3.47 ± 0.37 ka [19: S3]. These results are consistent between excavation units and with respect to stratigraphic order, but they are clearly at odds with the AMS ages. Two issues arise: how secure are the OSL ages in relation to the assumed cultural stratigraphy, and how could AMS ages on charcoal be so much younger?

Dewar et al. [19] chose the single-grain OSL method because of stratigraphic evidence of disturbance by termites. The extent of scatter in the distribution of OSL results for each sample indicated that disturbance had caused "significant contamination" [19: S3 p.7]. It was argued, nonetheless that, "from an archaeological standpoint, the most important feature to note is that the vast majority of grains in each sample belong to a single D_e population [i.e. a population defined by equivalent dose values]. For five of the six samples more than two-thirds of the grains in each sample formed a single D_e population" (19: S3 p.7), assumed to represent the host sediments; i.e. the dominant sediments assumed to represent the original deposits of the layer in question. An OSL age for Layer 2/3A, 930 ± 90 y B.P. was consistent with stylistic ages on artefacts from that level, "providing confidence in the accuracy of the [OSL] ages for the lower levels." In addition, it was thought highly likely, "that the artifacts excavated from these layers are contemporaneous with these grains [the host sediments] …and were not incorporated at

a later date" [19: S3 p.7], although the grounds for that view were not disclosed. Conversely, the AMS charcoal samples were thought intrusive in layers 4 and 5. The samples were not obtained from observed termite features, but there may have been "other burrows now completely consolidated, which have moved material downward," [19:S2 p.5]. It was suggested that termites "would likely move the charcoal along, as this would be a primary source of moisture; moisture management is the prime reason for termite burrowing." [19: S3 p.7]. No evidence is adduced for this proposition but its implication is that only charcoal was selected and displaced downward by termites, while the other archaeological components remained in primary association with the host sediments and were dated accurately by OSL.

Our argument also begins with the bioturbation issue. Termites excavate underground chambers connected to the surface by burrows or ventilation funnels and Madagascar also has insectivorous mammals that lead largely subterranean lives in burrows, notably the mole tenrecs (Oryzorictes). Dewar et al. concede that "it is likely that the [OSL] sample tubes may have penetrated some of the termite burrows" [19: S3 p.7], especially in layers 4 and 5 [19 S3: p.5] which were "penetrated by large later termite burrows" [19: 12585]. The extent of disturbance can be estimated through the proportion of measured quartz grains in minor D_e populations that did not belong to the host sediments. The lowest proportion is ~13% for sample ANJA K 4A/B, and it is 28-40% for the other five samples [19: Table 6]. In other words, about a third of the sediment in most samples had originated somewhere else than in the level occupied by the host population, and generally the minor components were younger, indicating downward displacement. Dewar et al. [19: S3 p.7], calculated that the 32.5% of minor D_e population grains in ANJA K3/A would provide an age of ~600 y B.P., compared to about 1330 y B.P. for the host sediment. Inspection of D_e values for minor grain components [19: Figure S3] suggests ages of ~1000 y B.P. or younger for samples ANJA J4A/B (2700 y B.P. on host sediments) and ANJA K 5A/B (3470 y B.P. on host sediments) and a somewhat younger age than the primary value in ANJA J 5B/C. Conversely, some older sediment than in the main population of grains is apparent in ANJA J2/3A and ANJA K4A. Consequently, although the dominant D_e populations at Lakaton'i Anja "indicate that the greatest proportion of grains in each sample are in primary context," [19: S3 p.7] there was also significant sedimentary disturbance by bioturbation in which charcoal and other cultural material could have been moved, especially downward.

On the existing data it is impossible to demonstrate that the stone artifacts and midden were associated exclusively, or even at all, with the host sediments. If charcoal was intrusive in layers 4 and 5, then bone, shell and stone probably were as well. Dewar et al. [19: S4 p.8] record local pottery sherds carried below layer 2 in termite burrows; a chlorite schist vessel sherd, dating <1000 y B.P. in layer 3B, and a dark glass bead of a type dating <1250 y B.P. (probably 950-700 y B.P., 19: S5 p.9) in layer 5 [19: 12587]. The stratigraphic distribution of cultural material suggests layers 1, and probably 3, were habitation floors with continuous deposits, while charcoal, bone and stone occurred as discrete concentrations in layer 4 and only as occasional fragments in layer 5. As the bone in the site was from small mammals, birds and fish, and as pieces of flaked stone in Lakaton'i Anja "are less than 2 cm in length" [Dewar et al. p.12585], it was all potentially susceptible to movement through bioturbation. The stratigraphic distribution of smaller stone flakes is weighted toward layers 4 and 5 [19: 12587], perhaps reflecting accumulation in termite burrows in the lower layers where further downward movement was precluded. In any event, the proposition that all classes of material were liable to be moved by bioturbation at Lakaton'i Anja, particularly downwards by gravity, constitutes

a simpler explanation of the stratigraphical distribution of cultural material than that the flaked stone and midden was in its original deposition, while charcoal was later moved by thirsty termites into coincidental association with it.

Differences by stratigraphic position in the type or technology of material culture at Lakaton'i Anja could contradict this differential displacement hypothesis; for example, if there were different technical traditions evident in the chert flake industry between layers. Coarse chert flakes were distributed fairly evenly through the stratigraphy although they were slightly more frequent in layers 1 and 5 [19: Table S2]. With little evidence of their production, no stratification in technical tradition was recorded and analysis focused on showing that "this is a stone tool industry created by people" [19: S2 p.5]. A similar argument [19: 12586], that a very small quantity (total of 0.11 g) of fine red chert was found exclusively in layers 4 and 5, is contradicted by red chert in layer 3 [19: S2 p.5]. Quartz crystal was said to be found "predominantly from layers 4 and 5" [19: S2 p.5], implying that it also occurred in upper layers. Little can be read either way into this evidence because the stratigraphic distribution of scarce items might reflect only the sampling effect of small excavations in a relatively large site (approximately 70m^2 floor area). Nevertheless, the data do not support suggestions of temporal change in material sources.

Older occupation in the site could also be inferred if it was apparent that the stone tools belonged to a tradition that had disappeared before the deposition of the upper layers at Lakaton'i Anja. Analysis of the flake tools, seemingly from layers 4 and 5 primarily, suggested several retouched blades and crescentic and trapezoidal forms "similar to those from Ambohiposa." [19: S2 p5]. There, however, such flakes were rejected as typologically defined "blades", "crescents" and "trapezes", and it was emphasized that "backed microblades, retouched points, or multiply notched tools that would be convincing to all stone tool specialists are not present" [19: S1 pp 2-3]. Dewar et al. [19: 12583-4] offer a rightly cautious appraisal of the evidence. The Madagascan industry is literally but not typologically microlithic; there are resemblances with other forager lithic assemblages located from southern Africa to South Asia, although not Southeast Asia, but there are no specific links with types in the Later Stone Age (LSA) microlith traditions of mainland East Africa. Recent linkage of the Madagascan and mainland LSA industries is advanced speculatively through a "lack of formal types" [24: 201], upon which there is little to hang an empirical case, and none is made. If there is a microlithic industry of East African LSA type in Madagascar then a demonstrated association of LSA lithics with ceramics dating ~1500-1000 y B.P. at Ukunji Cave on Mafia Island and late radiocarbon ages, <1100 y B.P., on assemblages in the lowest layer at Ambohiposa [19: 12585], do not support an inference of mid-Holocene antiquity at Lakaton'i Anja.

In summary we agree with Dewar et al. [19], that OSL ages on the dominant quartz grains provide a plausible chronology of the dominant sediments in Lakaton'i Anja, but we reject the inference that those ages refer necessarily to the deposition of archaeological material in the lower layers. We accept the probability that the radiocarbon ages represent displacement of charcoal samples from higher layers but argue that all of the relatively sparse archaeological material in the lower layers could also have been introduced by the extensive bioturbation signalled, most especially, in the 13-40% of non-host grains in the OSL samples. The non-host sediments appear generally younger (<1000-600 y B.P.) than the host sediments (2700-4380 y B.P.). The age of occupation at Lakatoni Anja remains uncertain, but the nine radiocarbon ages obtained in 1986 and 2011 are in the range 900-1700 y B.P., all but one younger than 1500 y B.P.

As these are on charcoal unidentified to taxa, and therefore might contain old wood, they must be regarded as maximum age ranges.

Supplementary Information References

51. Burney DA (1993). Late Holocene environmental changes in arid southwestern Madagascar. *Quaternary Research* 40: 98-106.
52. Blench RM (1996). The ethnographic evidence for long-distance contacts between Oceania and East Africa. *The Indian Ocean in Antiquity*, ed Reade J (British Museum, London) pp 461-470.
53. Simons EL et al. (1995). AMS ^{14}C dates for extinct lemurs from caves in the Ankarana massif, northern Madagascar. *Quaternary Research* 43(2): 249-254.
54. Dewar RE (1995). Of nets and trees: untangling the reticulate and dendritic in Madagascar's prehistory. *World Archaeology* 26(3): 301-318.
55. Burney DA (1999). Rates, patterns and processes of landscape transformation and extinction in Madagascar. *Extinctions in Near Time: causes, contexts and consequences*, ed MacPhee RDE (Kluwer Academic-Plenum, New York) pp 145-164.
56. Hingston M, Goodman SM, Ganzhorn JU, Sommer S (2005). Reconstruction of the colonization of southern Madagascar by introduced *Rattus rattus*. *Journal of Biogeography* 32: 1549-1559.
57. Muldoon KM, Simons EL (2007). Ecogeographic Size Variation in Small-Bodied Subfossil Primates From Ankilitelo, Southwestern Madagascar. *American Journal of Physical Anthropology* 134: 152-161.
58. Walsh MT (2007). Island subsistence: hunting, trapping and the translocation of wildlife in the western Indian Ocean. *Azania* 42(1): 83-113.
59. Blench R (2007) New palaeozoological evidence for the settlement of Madagascar. *Azania* 42: 69-82.
60. Godfrey LR, Jungers WL, Burney DA (2010). Subfossil lemurs of Madagascar. *Cenozoic Mammals of Africa*, ed Werdelin L, Sanders WJ (University of California Press, Berkeley) pp 351-367.
61. Muldoon KM, Goodman SM (2010) Ecological biogeography of Malagasy non-volant mammals: community structure is correlated with habitat. *Journal of Biogeography* 37(6): 1144-1159.
62. Tollenaere C et al. (2010). Phylogeography of the introduced species *Rattus rattus* in the western Indian Ocean, with special emphasis on the colonization history of Madagascar. *Journal of Biogeography* 37: 398-410.
63. Muldoon KM (2010). Paleoenvironment of Ankilitelo Cave (late Holocene southwest Madagascar): implications for the extinction of giant lemurs. *Journal of Human Evolution* 58: 338-352.

64. Lawler RR (2011). Historical demography of a wild lemur population (*Propithecus verreauxi*) in southwest Madagascar. *Population Ecology* 53(1): 229-240.
65. Gommery D, Ramanivosoa B (2011). Les lémuriens subfossiles dans le Nord-Ouest de Madagascar, du terrain à la diffusion des connaissances ou 15 ans de recherches franco-malgache. *Revue de Primatologie* 3: 1-15.
66. Crowley BE, Godfrey LR, Irwin MT (2011). A glance at the past: subfossils, stable isotopes, seed dispersal and lemur species loss in southern Madagascar. *American Journal of Primatology* 73(1): 25-37.
67. Serva M (2012). The settlement of Madagascar: what dialects and languages can tell us. *PLoS ONE* 7 (2) e30666.
68. Muldoon KM (2012). Early Holocene fauna from a new subfossil site: a first assessment from Christmas River, south central Madagascar. *Madagascar Conservation and Development* 7(1): 23-29.
69. Crowley BE, Samonds KE (2013). Stable carbon isotope values confirm a recent increase in grasslands in northwestern Madagascar. *The Holocene* 23(7): 1066-1073.
70. Ardalan A, Oskarsson M, van Asch B, Rabakonandriania E, Savollainen P. (2015). African origin for Madagascan dogs revealed by mtDNA analysis. *Royal Society Open Science* 2: 140552.
71. Rosenberger AL et al. (2015). Giant subfossil lemur graveyard discovered, submerged, in Madagascar. *Journal of Human Evolution* 81: 83-87.
72. Adelaar KA (2016). Austronesians in Madagascar: A critical assessment of the works of Paul Ottino and Philippe Beaujard. *Early Exchange between Africa and the wider Indian Ocean world*, ed Campbell G (Palgrave MacMillan, New York) pp 77-112.
73. Brucato N et al. (2016). Malagasy genetic ancestry comes from an historical Malay trading post in southeast Borneo. *Molecular Biology and Evolution* 33(9): 2396-2400.
74. Raison JP Vérin P (1967). Le site de subfossiles de Taolambiby (Sud-Ouest de Madagascar) doit-il être attribué à une intervention humaine? *Annales de l'Université de Madagascar. Série Lettres et Sciences Humaines*, 1967: 133-142.
75. Brock F, Higham T, Ditchfield P, Bronk Ramsey C (2010) Current pretreatment methods for AMS Radiocarbon dating at the Oxford Radiocarbon Accelerator Unit (ORAU). *Radiocarbon* 52(1): 103-112.
76. Hogg AG et al. (2013), SHCal13 Southern Hemisphere Calibration, 0-50,000 Years cal BP. *Radiocarbon* 55(4). DOI: 10.2458/azu_js_rc.55.16783

Fig A. Marked *Hippopotamus* bones from the main pit at Ambolisatra; vertebral neural spine (ANU 16-1) with possible upper and lower bite or gnawing marks from a rodent or tenrecid under normal light (a), and SEM (b-c); abraded radius (ANU 11-1) under normal light (d) and SEM (e, f); a rib (ANU 30-1) with scratches under normal light (g) and SEM (h-i).

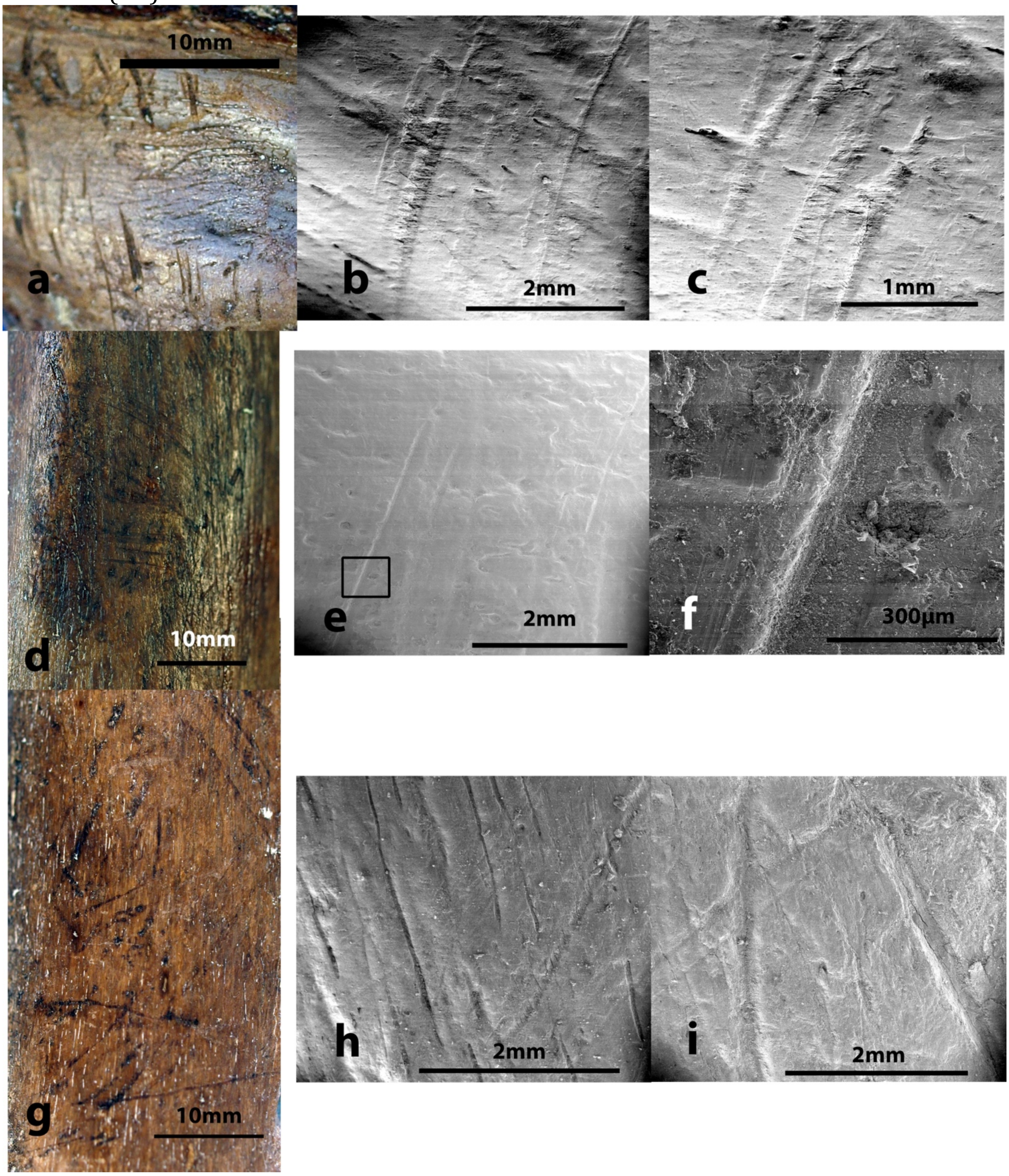

Fig B. Marked bones from Itampolo: *Cryptoprocta spelea* proximal femur (ANU 85-1) with scratches under normal light and SEM (a, b); *Hippopotamus* femur (ANU 54-1) with scoring mark under normal light and SEM (c,d); *Hippopotamus* ilium (ANU 55-1) under normal light and SEM images (e-h) showing multiple episodes of abrasion.

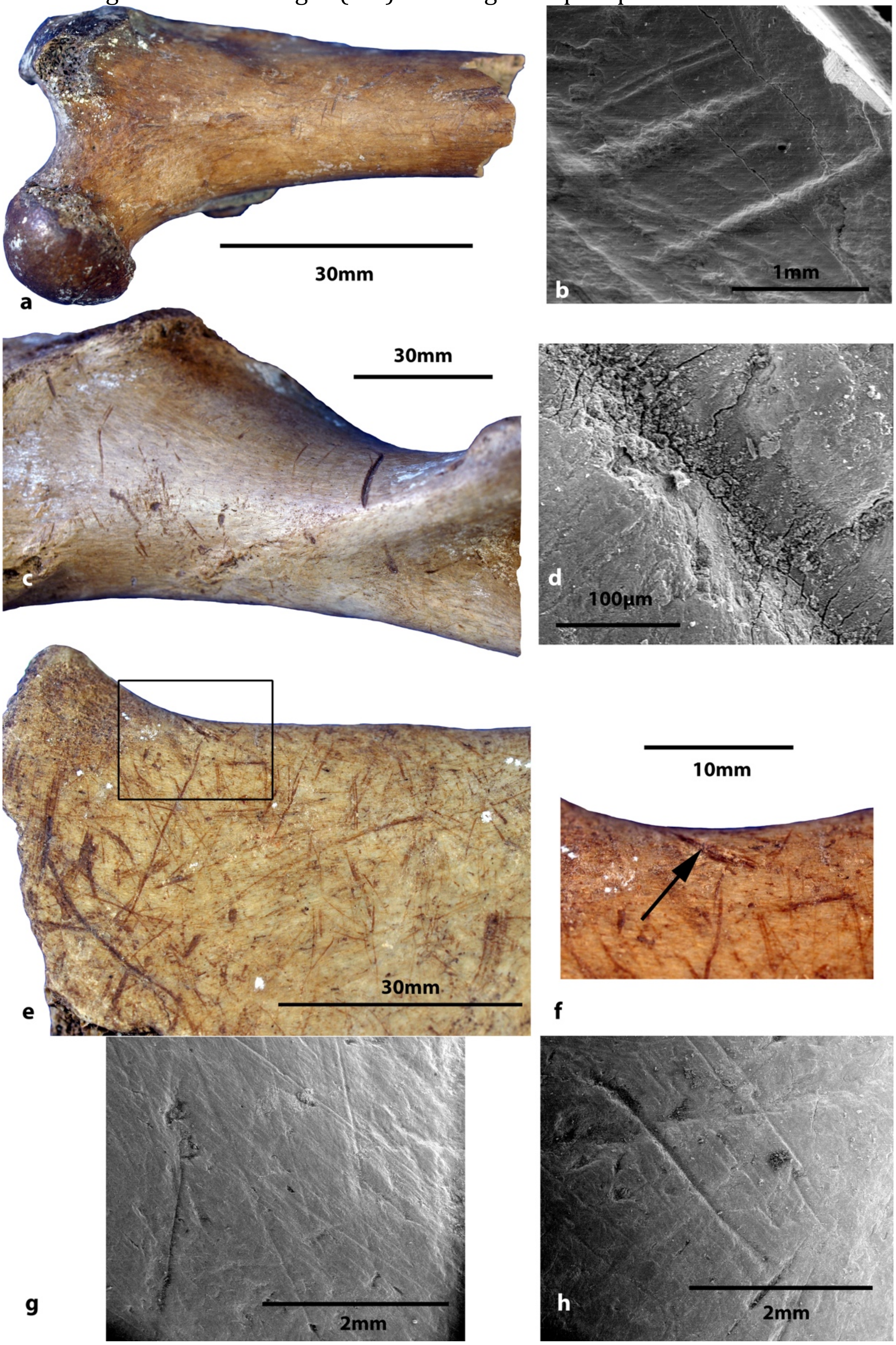

Fig C. Cutmarked *Propithecus verrauxi* bones from Taolambiby; a rib (ANU 061) under normal light and SEM (a-c); distal femur (ANU 069) under normal light and SEM (d, e); proximal tibia shaft of ANU 069 with spiral fracture and chatter-marks under SEM and normal light (f, g); ulna (ANU 055) with cut-marked olecranon process in lateral and dorsal/cranial views under normal light and SEM (h-j).

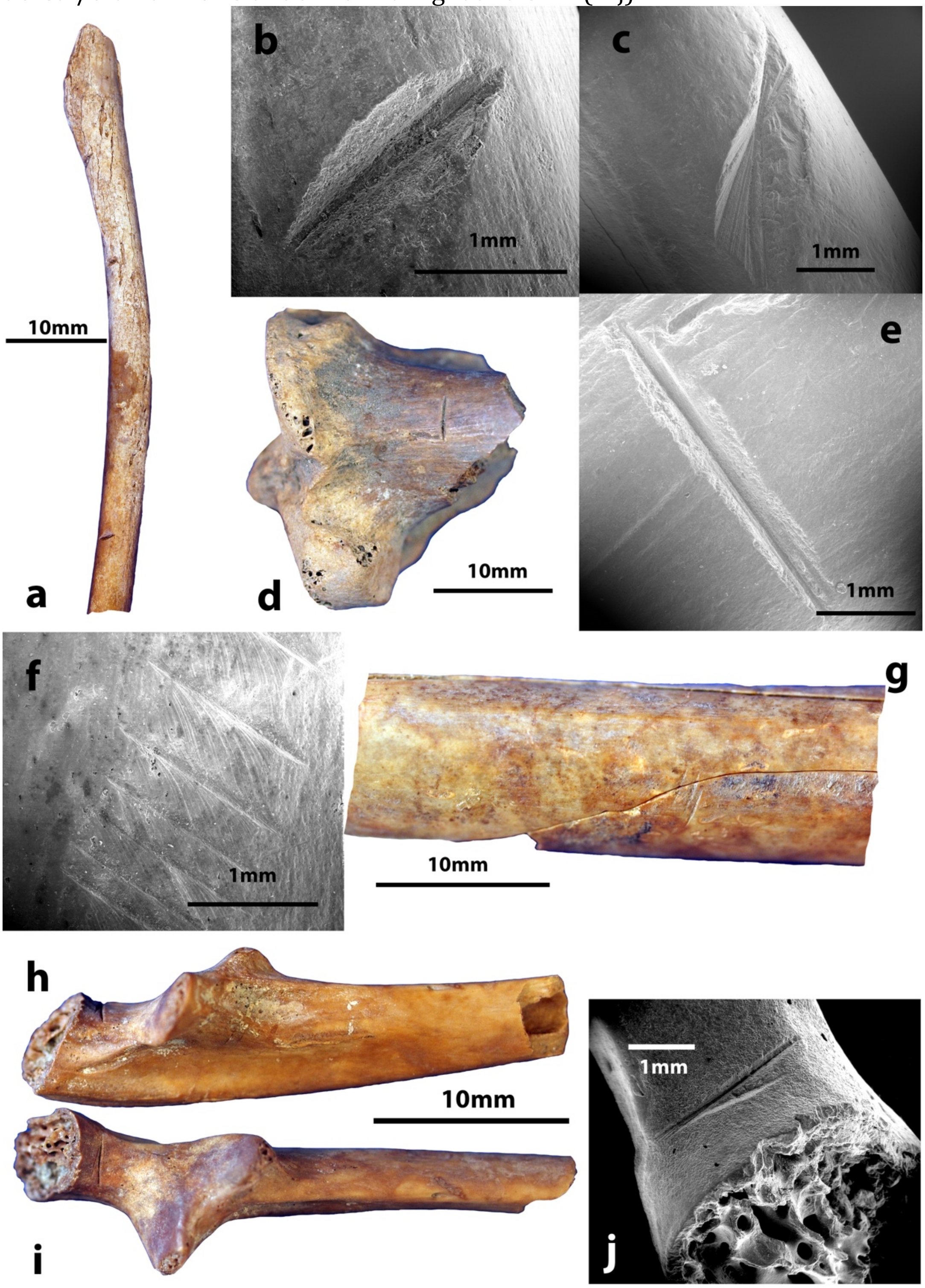

Fig D. Cutmarked bones from Taolambiby under normal light and SEM. *Propithecus verrauxi* limb bone shaft (ANU 071) (a-d); *P. verreauxi* clavicle (ANU 085) (e-g); and contrasting natural bone damage under SEM and normal light on *Pachylemur insignis* humerus (ANU 119) (h-j).

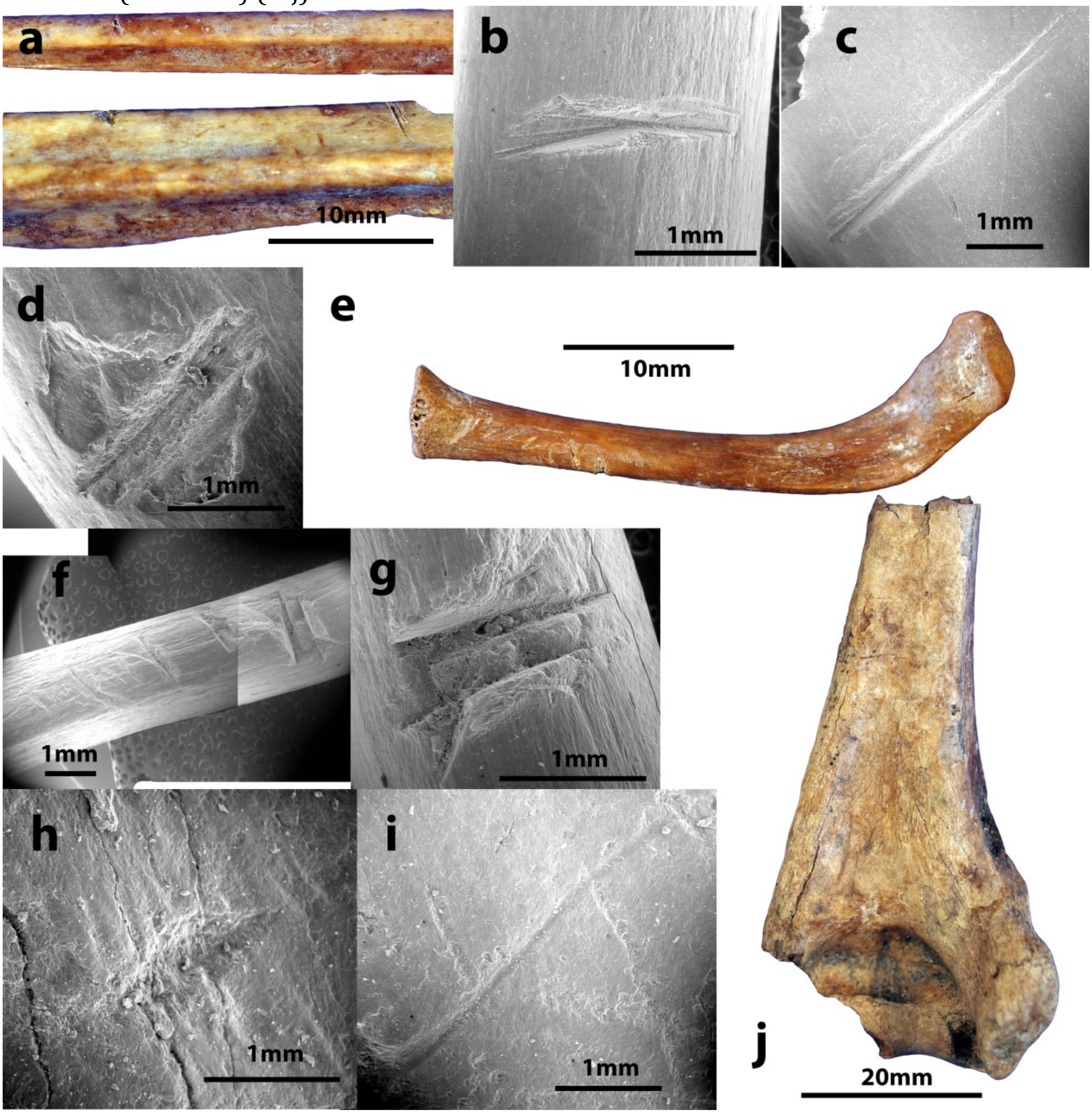

Fig E. Cross sectional profiles (locations in images shown by red lines), and associated graphs by MZ16 stereoscopic microscope through marks on the surface of *Palaeopropithecus ingens* limb bones from the Methuen collection; (a), OUM 14346b; (b-d) various locations on OUM 14342A; (e), *P. verreauxi* metapodial (ANU 043) Taolimbiby excavations with multiple cutmarks (Text Fig 4, g-h).

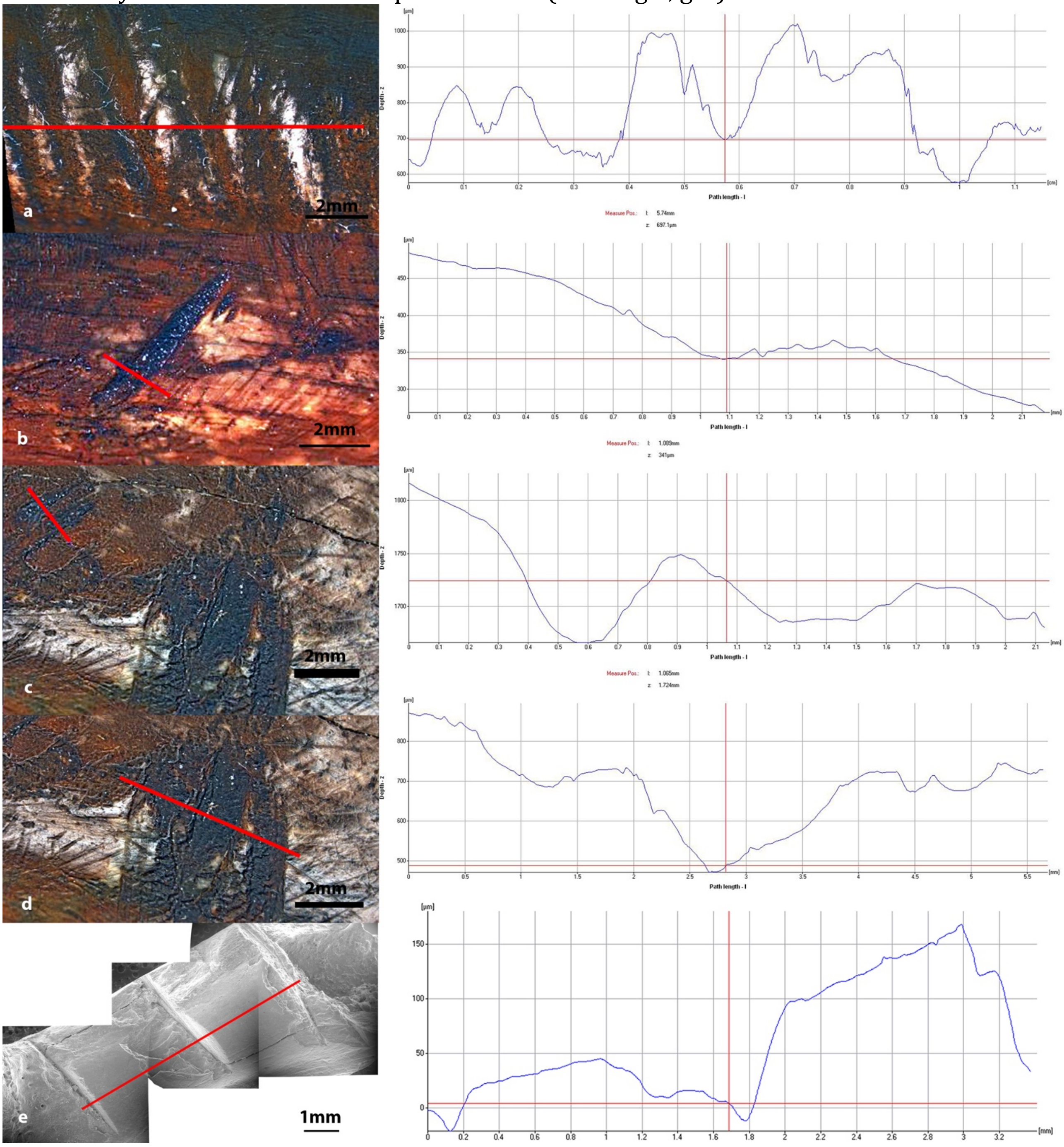

Fig F. Extinct lemur metapodial (ANU 103) from Taolambiby with possible rodent gnaw marks under normal light and SEM (a-c), with close-up (d) of gnawing on distal end; distal tibiotarsus of *Aepyornis maximus* (ANU 117-1) from Taolambiby showing probable bite-marks with associated crush fracture (e).

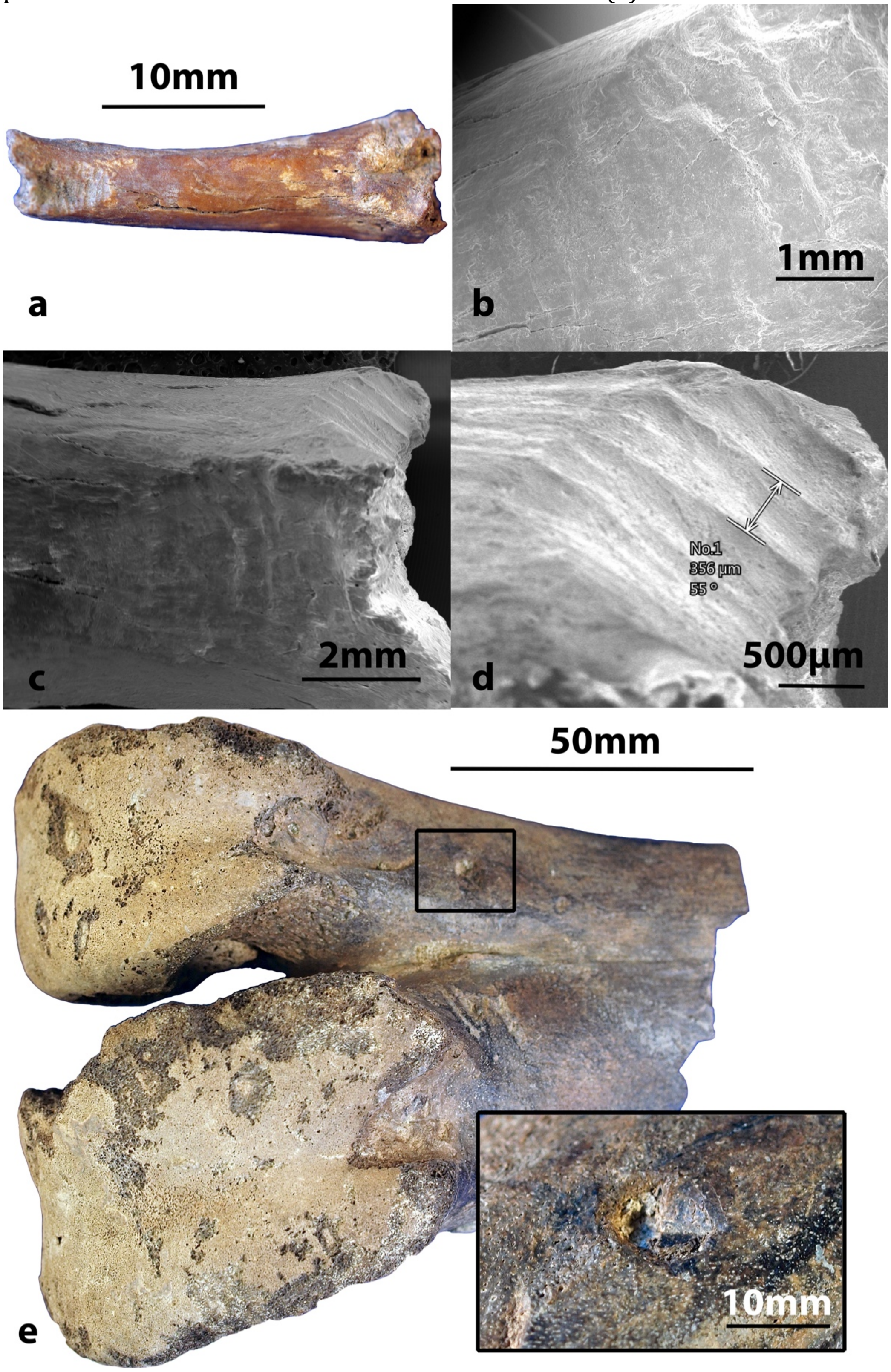

Fig G. *Crocodylus niloticus* vertebra (ANU 28-1) (a) from Ambolisatra and (b), *Hippopotamus* femur (ANU 126-1) from Taolambiby; both showing modern excavation damage.

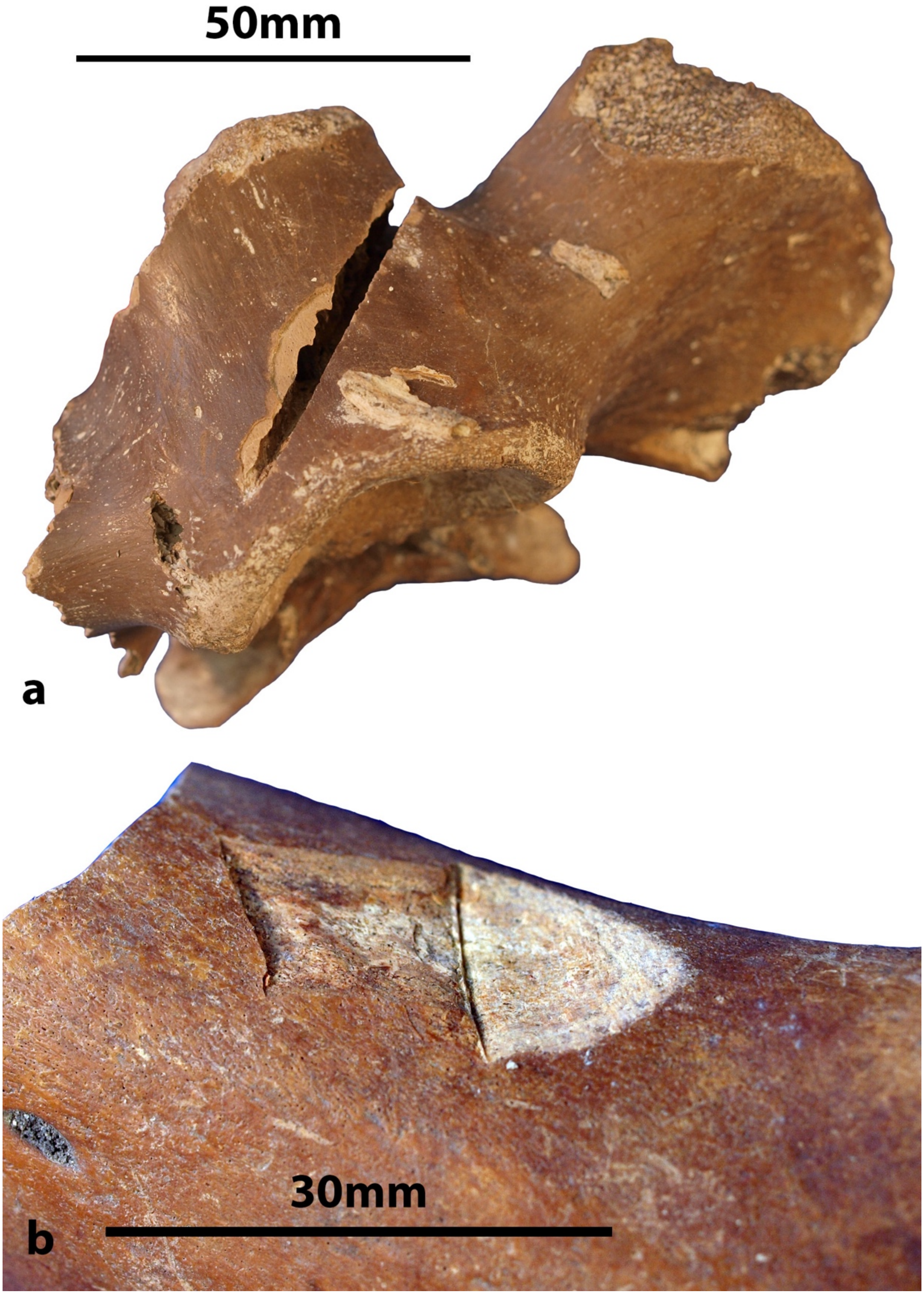

Fig H. Examples of chop-marked *Hippopotamus* femora from southwest Madagascan sites in the Grandidier collection (NMHN): (a) and (d), top and side views of MAD8398; (b) and (e), from Lamboharana 1901, MAD1710; (c) and (f), Ambolisatra 1898, MAD1711. Epiphyseal pieces from Lamboharana 1901; (h) and (i), MAD 1708 left and MAD 1709 right, in each image; (j) left femur MAD8392.

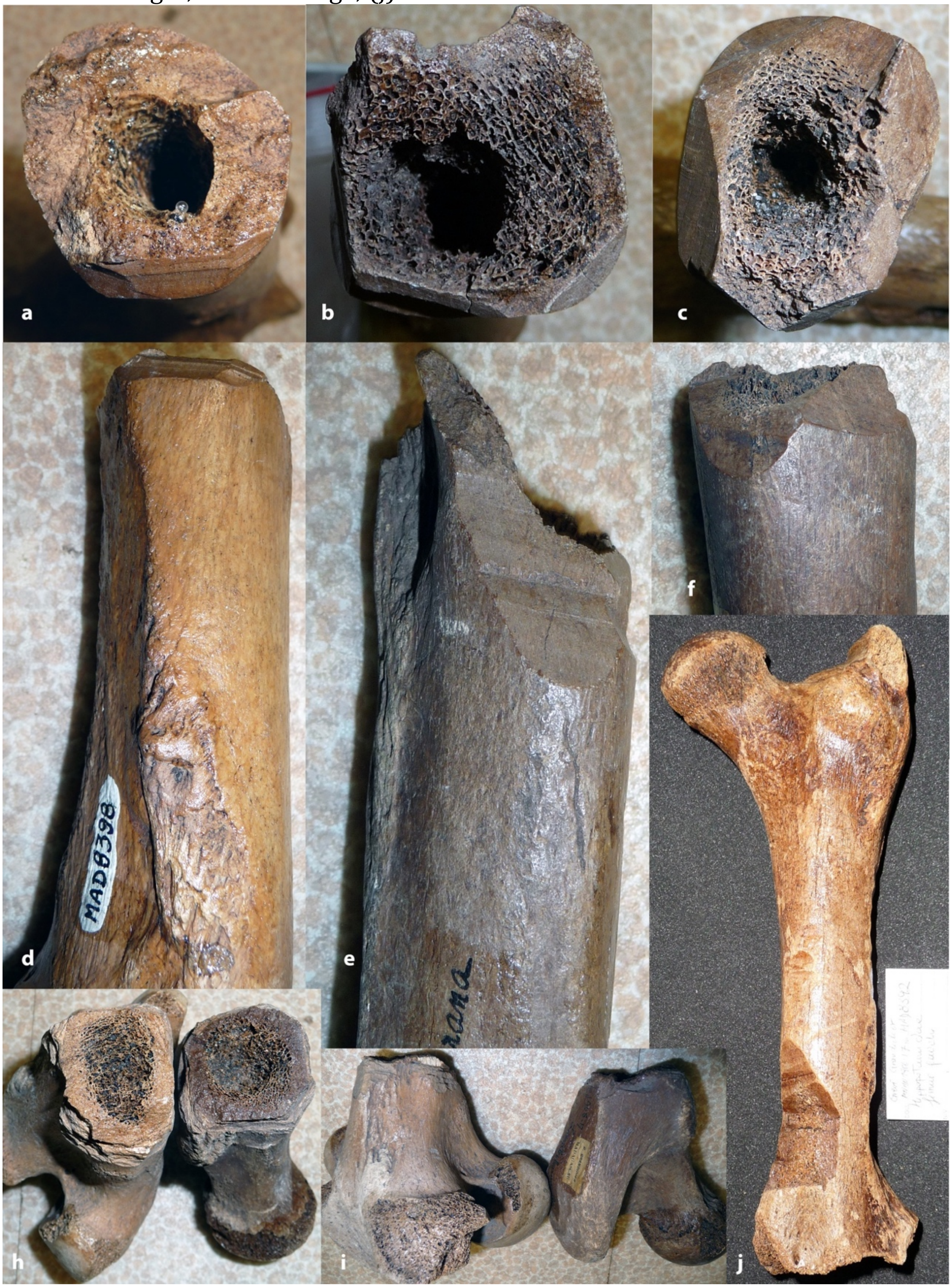

Fig I. Sedimentary core from Ambolisatra showing drying phase from earlier than 1800 y B.P up to about 1600 y B.P. associated with increased *Sporomiella*, indicative of increased herbivory, and increasing micro-charcoal 1600-700 y B.P. with major increase in macro-charcoal beginning about 1000-700 y B.P (age results in Table F).

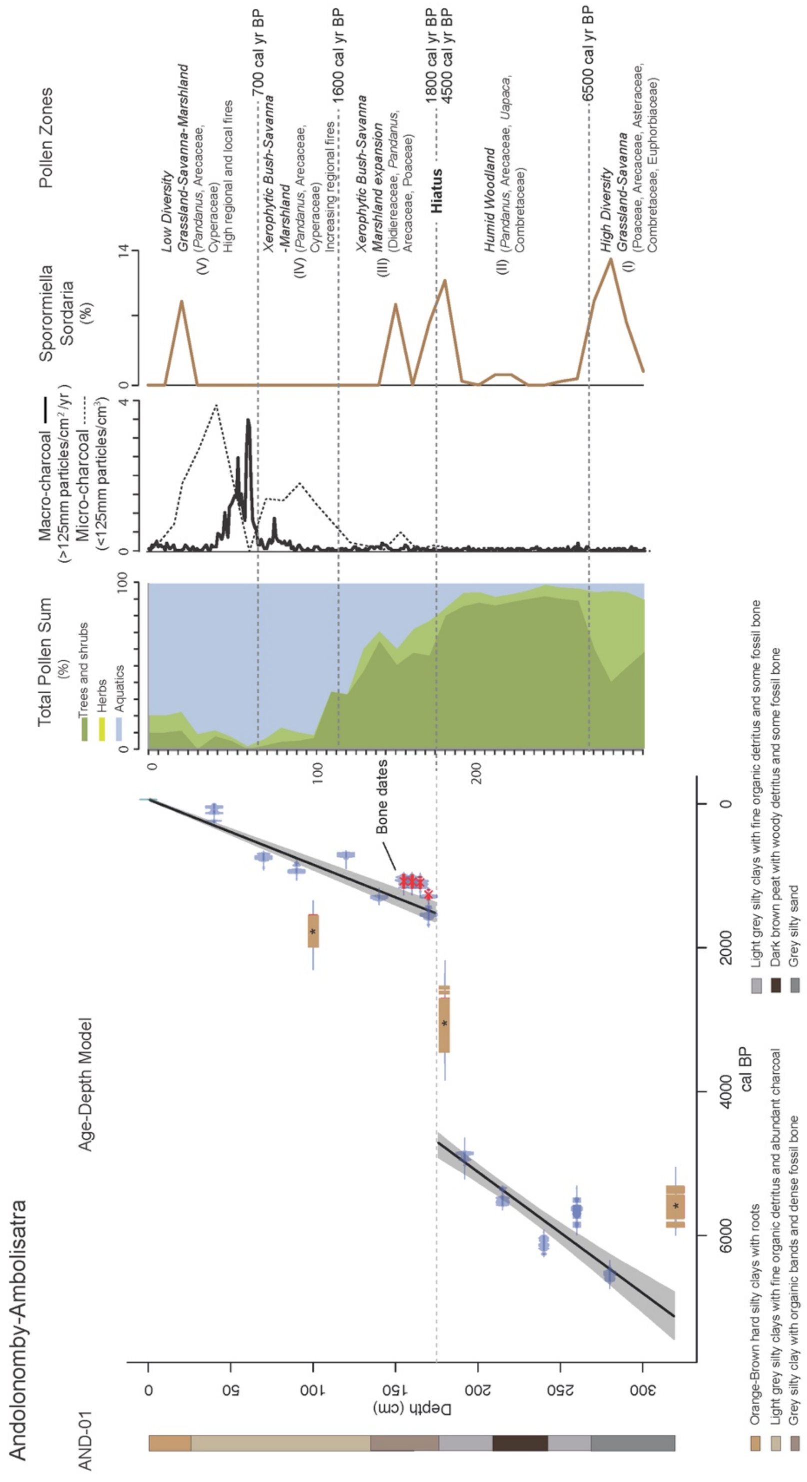

Fig J. Frequency of charcoal particles (>125 microns) per cm^2/year from Taolambiby Area 3 stratigraphy against radiocarbon dates all sites in this study (Text Fig 5), showing that continuous curve of charcoal increase begins contemporaneously with youngest dates on megafauna and earliest on cutmarked bones of extant fauna, ~900 cal B.P.

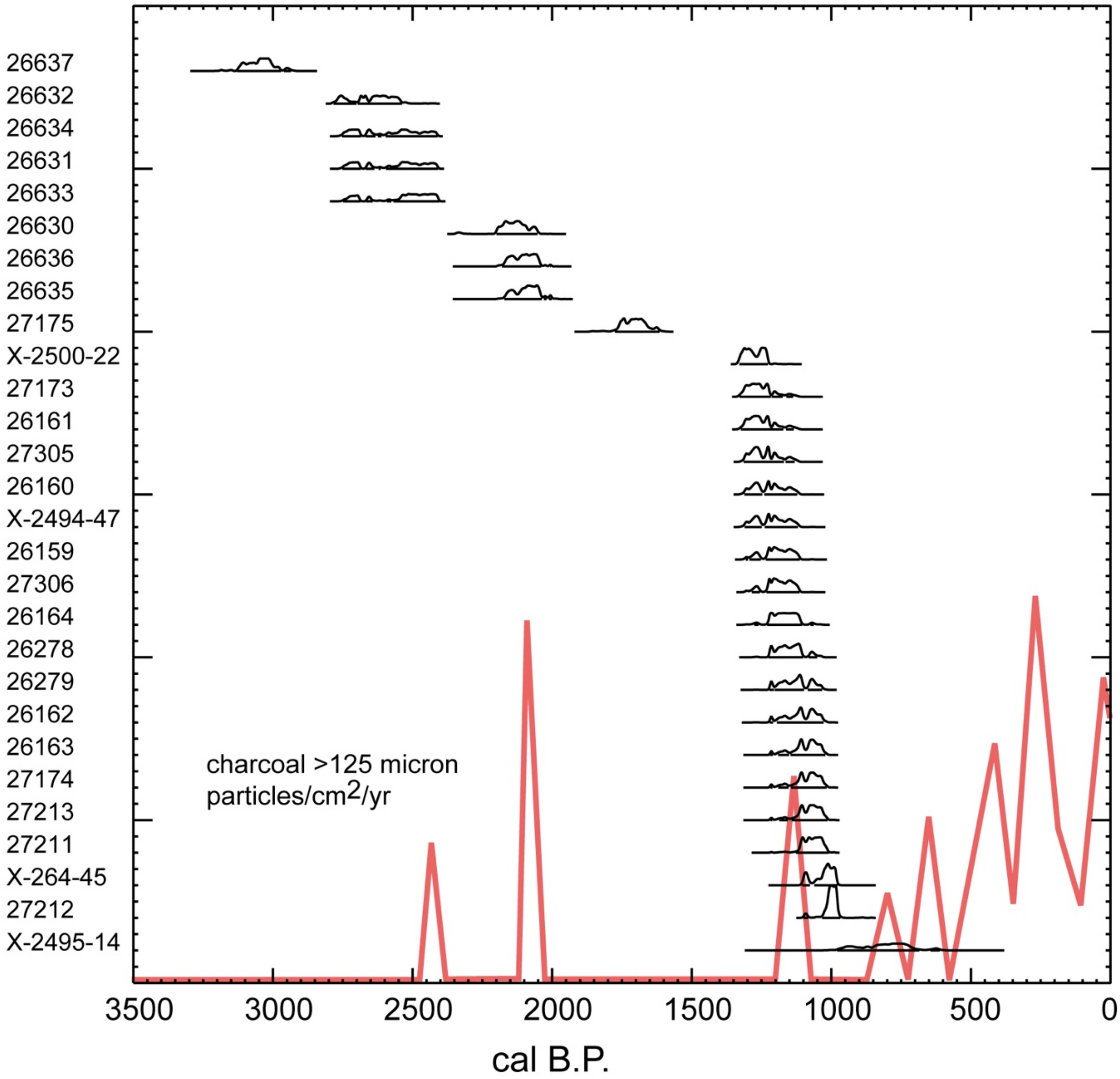

Fig K. Plan of the Taolambiby site showing areas excavated in this research.

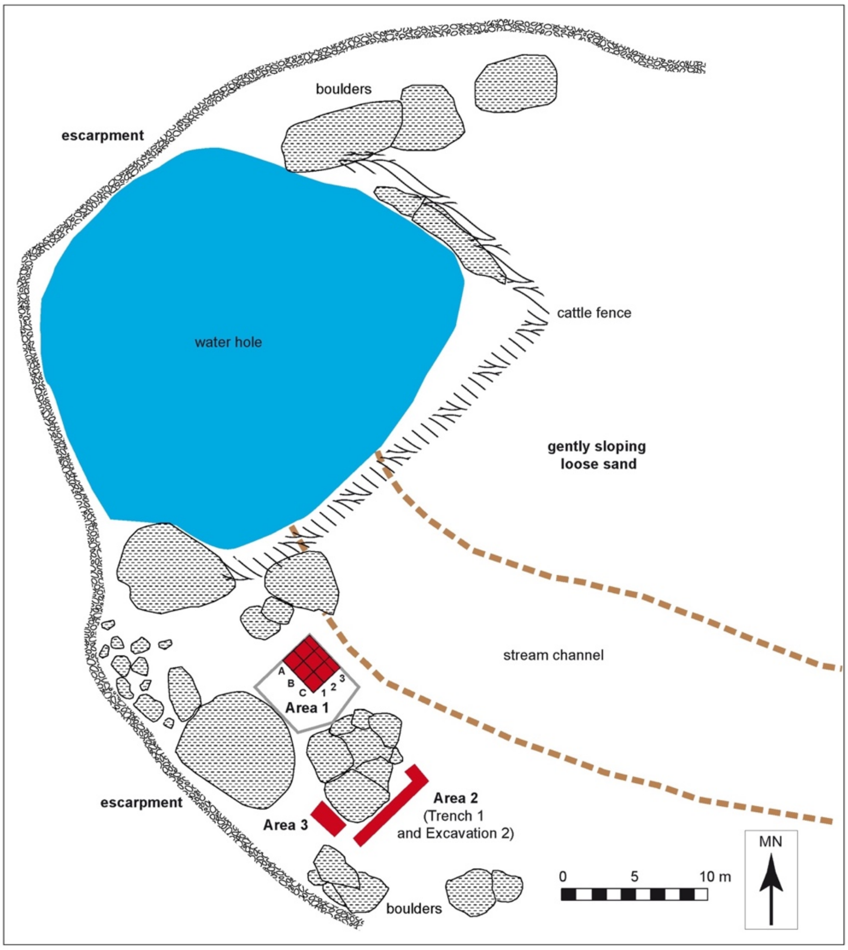

Fig L. Cross-section of excavation Area 2 at Taolambiby. The sandstone shown above the sedimentary layers represents a stone buttress rising on the far side of the excavation.

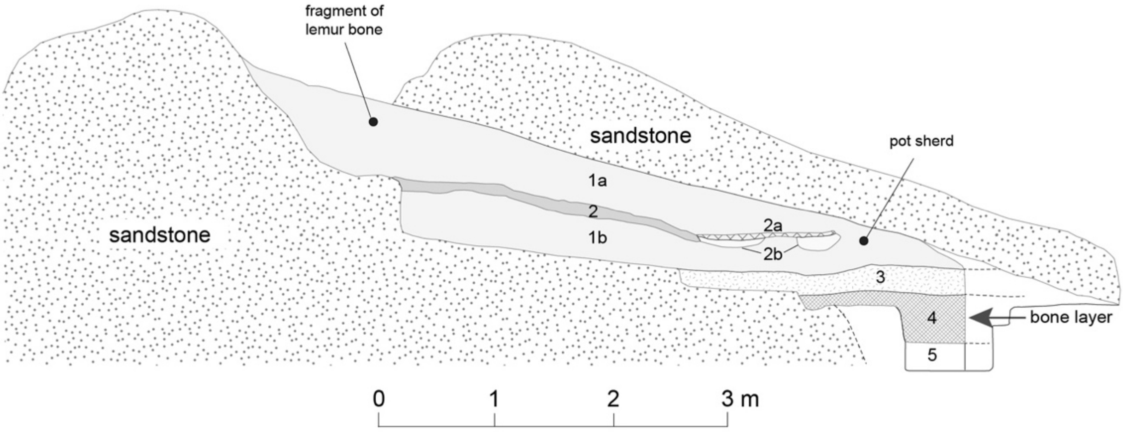

Fig M. Plan of the Ambolisatra site showing areas excavated in this research.

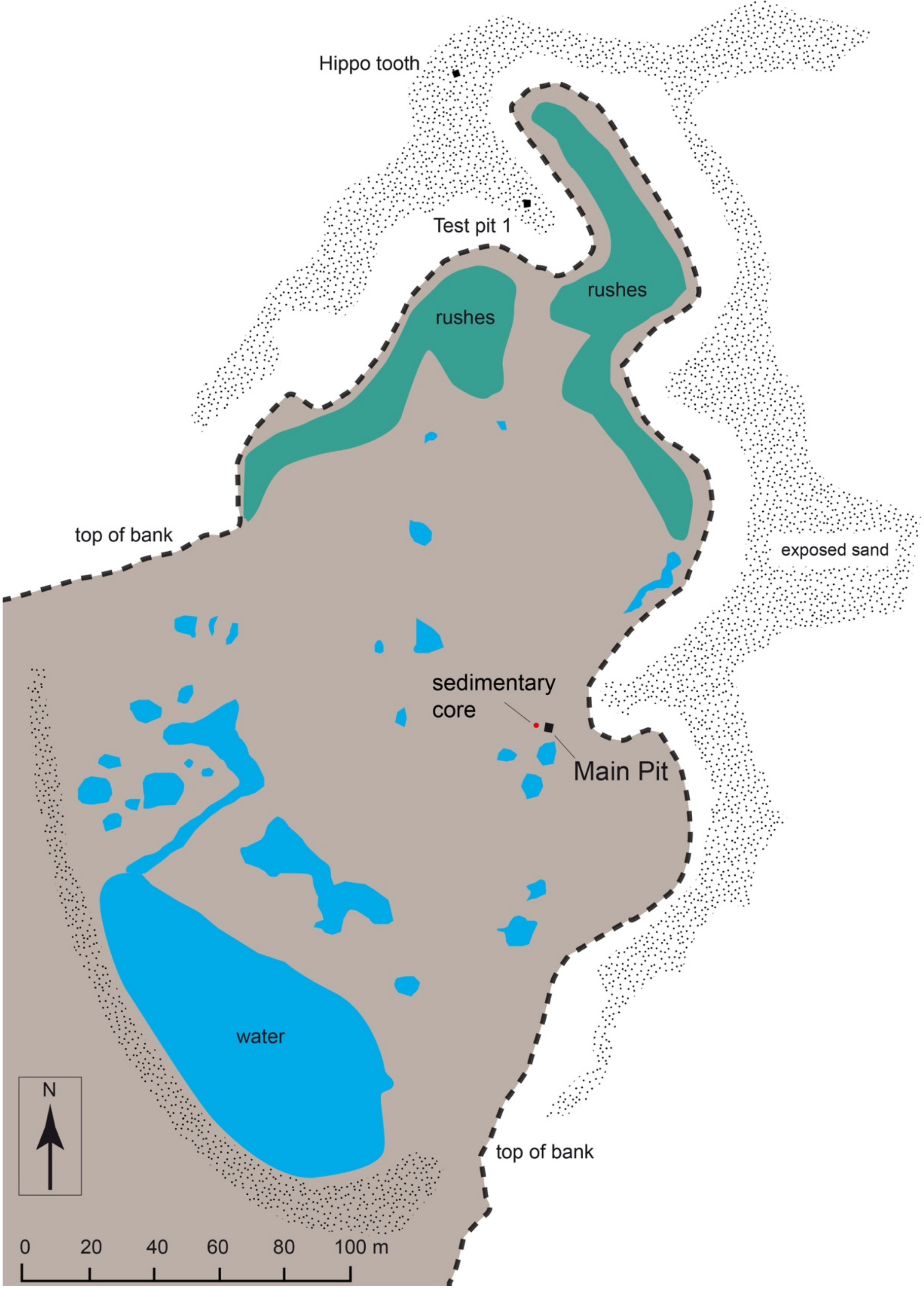

Fig N. Plan of the Itampolo site showing areas excavated in this research.

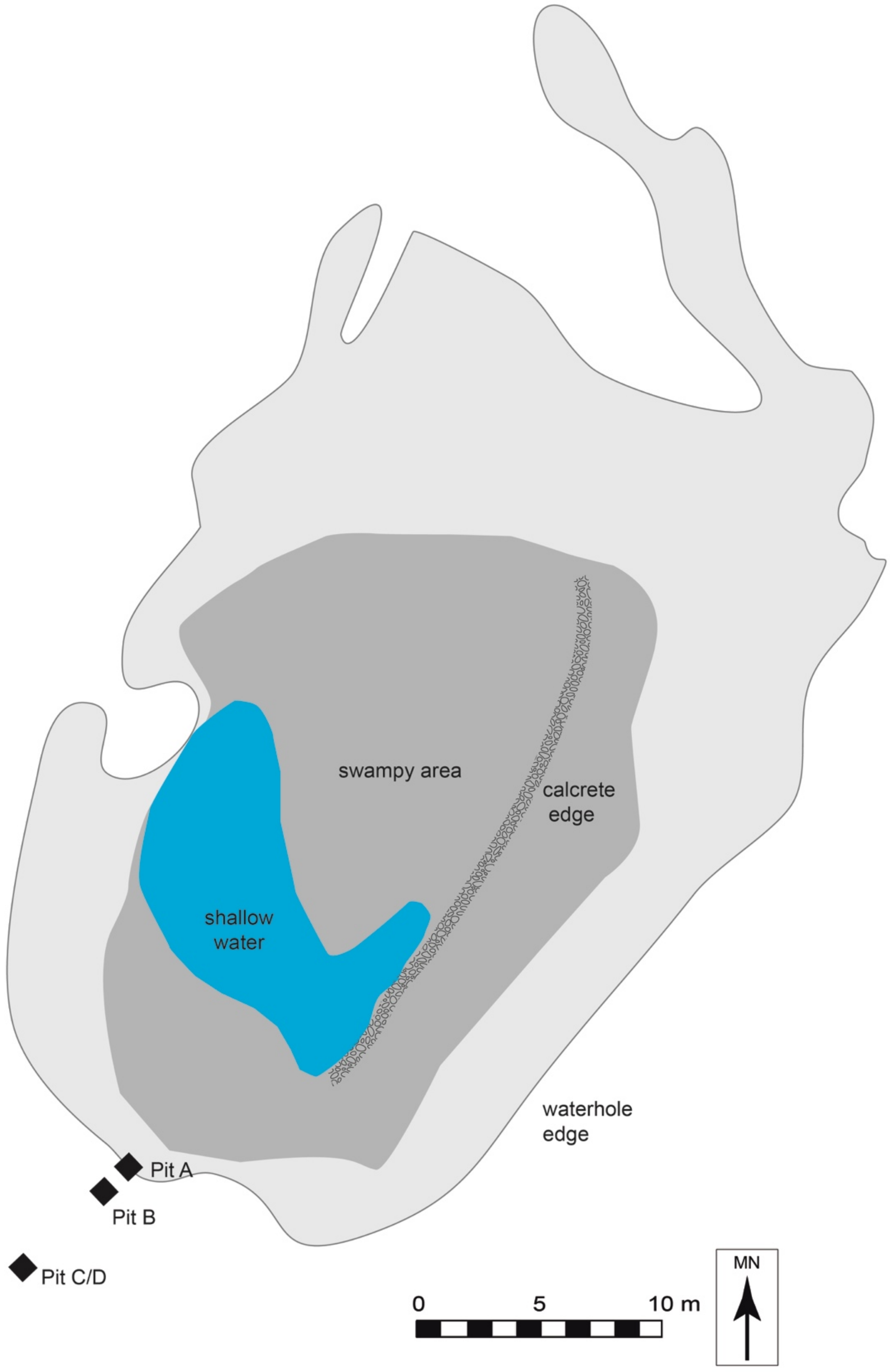

Table A. Incidence of cutmarks (C) on excavated fauna (ANU 2011 excavations) by NISP and MNI.

TAXA	Ambolisatra		Itampolo		Taolambiby		Totals		
MEGAFAUNA EXTINCT	NISP (MNI)	C	NISP (MNI)	C	NISP (MNI)	C	NISP	C	C% NISP
Giant Lemurs	5 (1)	-	21 (6)	-	20 (6)	-	46	-	-
Cryptoprocta spelea	-	-	3 (2)	-	-	-	3	-	-
Hippopotamus	220 (6)	-	625 (15)	1	269 (7)	1	1114	2	0.17
Giant Tortoise	-	-	11 (6)	-	213 (4)	-	224	-	-
Crocodile*	243 (6)	-	19 (3)	-	77 (2)	-	339	-	-
Elephant bird	30 (1)	-	23 (5)	-	8 (3)	-	61	-	-
EXTANT		-	-	-		-		-	-
Cryptoprocta ferox	-	-	-	-	10 (2)	5	10	5	50.00
Propithecus verreauxi	-	-	-	-	224 (7)	32	224	32	14.28
Miscell. Mammal	-	-	-	-	666 (19)	-	666	-	-
Miscell. Bird	-	-	-	-	23 (9)	-	23	-	-
NISP (MNI)	498 (14)	-	702 (37)	-	1510 (59)	-	2710	39	-

Table B. Characterisation of damage on distal end of OUM 14342A with reference to relevant criteria in Dominguez-Rodrigo et al. [42]*

Criteria	Trampling	Cutmark (unretouched flake)	Cutmark (retouched flake)	OUM 14342A distal
**Mark trajectory**	Curved/sinuous70 %	Curv/sin 7%	Curv/sin 3%	Curv/sin 60%
**Mark orientation**	Oblique 82%<br>Perpendicular 8%	Obliq. 61%<br>Perp. 39%	Obliq. 97%<br>Perp. 3%	Obliq. 80%<br>Perp. 5%
**Groove cross-section**	Broad V/U 96%	Broad V/U 3%	Broad V/U 94%	Broad V/U 90%
**Shoulder effect**	Present 6%	Present 33%	Present 74%	Present 10%
**Shoulder flaking**	Present 3%	Present 15%	Present 51%	Not present
**Overlapping striae**	Present 80%	Present 13%	Present 0%	Present 50%

*Some criteria do not distinguish between causes, e.g. microabrasion is present at rates of 98-100% in each category.

Table C. Bones (n= 449) in Grandidier Collection MNHN examined for damage (n).

Genus	Radii, Ulnae, Fibulae	Tibiae	Femora	Humeri	MNE Total
Archaeolemur	2R	7(1)	18(2)	8	35(3)
Hadropithecus				2	2
Palaeopropithecus	6R	6	2	20(4)	34
Lemur Total	6R, 11U, 2F	4	18(1)	12	53(1)
Hippopotamus	R&U 86(1)	63(2)	115(22)	61(5)	325(30)

Table D. Summary of the fossil material excavated at Taolambiby, Itampolo and Ambolisatra as part of this study. † indicates that taxa are extinct.

Identification	Taolambiby		Itampolo		Ambolisatra	
Mammalia	MNI	NISP	MNI	NISP	MNI	NISP
†Archaeolemur cf. majori	2	3	-	-	-	-
Cheirogaleus cf. major	1	2	-	-	-	-
Cryptoprocta cf. ferox	2	10	-	-	-	-
†Cryptoprocta spelea	-	-	2	3	-	-
Echinops sp.	1	2	-	-	-	-
Eulemur cf. fulvus	1	3	-	-	-	-
Geogale cf. aurita	1	1	-	-	-	-
Hapalemur cf. simus	1	8	-	-	-	-
†Hippopotamus cf. lemerlei	7	269	15	625	6	220
Lemur cf. catta	2	28	-	-	-	-
Microcebus sp.	1	3	-	-	-	-
Microgale cf. thomasi	1	1	-	-	-	-
†Pachylemur cf. insignis	4	10	-	-	-	-
†Palaeopropithecus	-	-	1	2	-	-
Propithecus cf. verrauxi	7	224	-	-	-	-
cf. Setifer	2	19	-	-	-	-
cf. Eliurus minor	1	2	-	-	-	-
rat-sized rodent	1	37	-	-	-	-
mouse-sized rodent	1	1	-	-	-	-
Tenrec ecaudatus	3	63	-	-	-	-
unknown mongoose	1	2	-	-	-	-
indet. extant lemur	NA	194	-	-	-	-
†indet. extinct lemur	NA	7	5	19	1	5
indet. small mammal	NA	300	-	-	-	-
Reptilia						
†Geochelone	4	213	6	11		
Crocodylus niloticus	2	77	3	19	6	243
Aves						
Anatid (duck)	1	1	-	-	-	-
Columbidae cf. Geopelia striata	1	1	-	-	-	-
Tytonidae (owl)	1	1	-	-	-	-
Passerine	1	3	-	-	-	-
Columbidae cf. Streptopelia	1	1	-	-	-	-
cf. Centropus	1	2	-	-	-	-
Columbidae	1	1	-	-	-	-
Centropus cf. toulou (ground cuckoo)	1	1	-	-	-	-
misc bird	1	12	-	-	-	-
†Aepyornis sp.	3	8	5	23	1	30
**Totals**	**59**	**1510**	**37**	**702**	**14**	**498**

Table E. Occurrence of extinct large fauna (> 10 kg bodyweight): column a= previously recorded (x), and column b= found in ANU 2011 excavations (X) reported here.

Extinct fauna	Adult weight (kg)	Ambolisatra		Taolambiby		Itampolo	
**Lemurs**		a	b	a	b	a	b
Palaeopropithecus ingens	42 - 45.4	x		x			X
Palaeopropithecus nov. sp. ?							
Mesopropithecus globiceps	9.4 - 11			x			
Archaeolemur majori	13.9 - 18			x	X		X
Archaeolemus edwardsi	24.5 -26.5						
Hadropithecus stenognathus	27.1 - 35						
Megaladapis edwardsi ?	75.4 - 85	x		x			
Medaladapis madagascariensis	38 - 46.5			x			
Pachylemur insignis	10 – 11.5	x		x	X		
Daubentonia robusta	13.5 – 14						
Extinct Lemur unidentified			X		X		
**Other taxa**							
Cryptoprocta spelea	17-20			x			
Plesiorycteropus madagascariensis	6-18	x		x			
Hippopotamus cf. *lemerlei*	180-275		X	x	X	x	X
Aepyornis spp.	300-500			x	X		
Mullerornis spp.	100-200	x					
Geochelone grandidieri or *abrupta*	150-250			x	X		X
*Crocodylus niloticus**	200-500			x	X		

*Crocodile is extirpated in Madagascar but extant in mainland Africa.

Table F. Ambolisatra/Andolonomby ages for samples in Fig I where calibrated ages are reported [18, 51]. * $\delta^{13}C$ values not calculated.

Sample ref.	^{14}C age	$\delta^{13}C$	Depth (cm)	Comments
Surface	Modern*	*	0	Assumed age
Wk-35193	94 ± 25	-16.4	40-41	Bulk sediment
Wk-38880	879 ± 29	*	69-70	Bulk sediment
D-AMS 006679	1063 ± 27	-14.7	89-90	Bulk sediment
B-24345	1890 ± 90	-24.1	90-100	Sediment (18, 51)
Wk-38881	843 ± 25	*	120-121	Bulk sediment
Wk-35194	1436 ± 25	-24.7	140-141	Bulk sediment
OxA-26104	1214 ± 24	-18.1	155	Lemur (extinct) metapodial
OxA-26102	1220 ± 24	-17.1	160	Hippo sp. humerus
OxA-26105	1239 ± 23	-18.13	165	Lemur (extinct) metapodial
OxA-26103	1417 ± 23	-18.14	170	Hippo sp. tooth
Wk-38882	1699 ± 25	*	170-171	Bulk sediment
B-23149	2915 ± 170	-24.5	180	Bone sp.? (18, 51)
D-AMS 006680	4370 ± 26	-26	192-193	Bulk sediment
Wk-38883	4810 ± 28	*	214-215	Bulk sediment
Wk-35195	5397 ± 27	-24.9	240-241	Bulk sediment
B-23148	4965 ± 70	-24.6	260	Bone sp.? (18, 51)
Wk-35196	5806 ± 27	-26.2	280-281	Bulk sediment

Table G. AMS Radiocarbon ages (ORAU) for material submitted in the current project (see Text Fig 5).

Site	Context	Identification/Element	OxA Number	CRA	13C	cal B.P. 95.4%
Ambolisatra	Main Pit bone layer	*Hippopotamus* sp., humerus	26102	1220±24	-17.10	986-1178
Ambolisatra	Main Pit bone layer	*Hippopotamus* sp., tooth	26103	1417±23	-18.14	1270-1315
Ambolisatra	Main Pit bone layer	Lemur (extinct sp.), metapodial	26104	1214±24	-18.10	982-1177
Ambolisatra	Main Pit bone layer	Lemur (extinct sp.), metapodial	26105	1239±23	-18.13	1004-1181
Itampolo	Andronovony, Well Pit	*Archaeolemur*, fibula	26106	1618±25	-19.26	1384-1535
Itampolo	Ankororohe, Square D	*Hippopotamus* sp., femur	26107	1883±25	-12.41	1714-1832
Itampolo	Ankororohe, Square D, 65-80 cm	*Hippopotamus* sp., tooth	26108	1271±24	-14.89	1068-1260
Itampolo	Ankororohe, Square D, 120-140 cm	*Hippopotamus* sp., tooth	26109	1376±24	-12.72	1185-1301
Itampolo	Ankororohe, Square D, 80-100 cm	*Hippopotamus* sp., tooth	26110	1576±25	-17.42	1360-1511
Itampolo	Ankororohe, Square D, 105-130 cm	*Hippopotamus* sp., tooth	26111	1305±23	-14.07	1093-1271
Itampolo	Andronovony, Well Pit	Tortoise (extinct sp.), carapace/plastron	26112	1835±38	-12.90	1609-1823
Itampolo	Ankororohe, Square D, 120-140 cm	*Palaeopropithecus*, skull	26113	1708±24	-19.01	1525-1693
Itampolo	Ankororohe, Square D, 60-100 cm	*Hippopotamus* sp., tooth	26208	1311±26	-15.15	1093-1273
Itampolo	Andronovony, Well Pit	*Hippopotamus* sp., skull, jugal	26209	1627±26	-14.10	1409-1537
Taolambiby	Area 1, Sq. A1, surface	cf. *Eulemur*, tooth	X-264-45	1064±30	-20.06	808-970
Taolambiby	Area 1, Sq. C00, Layer D, 10 cm	*Archaeolemur*, tibia	26159	1218±27	-21.36	983-1178
Taolambiby	Area 1, Sq. B0, Layer D, 10-15 cm	*Pachylemur insignis*, tooth	26160	1234±27	-20.81	992-1181
Taolambiby	Area 1, Sq. C1, surface	Crocodile (cf. *Crocodylus niloticus*), mandible	26161	1251±28	-18.14	1001-1259
Taolambiby	Area 1, Sq. B0, Layer D, 10-15 cm	*Propithecus verrauxi*, tympanic bulla	26162	1150±28	-19.07	956-1064
Taolambiby	Area 1, Sq. C0, 5-10 cm	*Propithecus verrauxi*, mandible	26163	1142±27	-18.82	937-1061
Taolambiby	Area 1, Sq. C0, 5-10 cm	*Propithecus verrauxi*, mandible	26164	1193±27	-19.38	964-1170
Taolambiby	Area 1, Sq. C1, surface	*Propithecus verrauxi*, tooth	26278	1175±26	-19.52	963-1069
Taolambiby	Area 1, Sq. C0, 10-15 cm	*Lemur* cf. *catta*, tooth	26279	1160±27	-20.23	961-1063
Taolambiby	Area 1, Sq. C1	*Hippopotamus* sp., metacarpal	X-2494-47	1231±27	-16.14	990-1181
Taolambiby	Area 1, Sq. C1 in grey sand	Charcoal	X-2495-14	810±100	-26.68	554-905
Taolambiby	Area 1, Sq. C2, surface	*Pachylemur* cf. *insignis*, anterior mandible	X-2500-22	1283±24	-21.79	1072-1265
Taolambiby	Area 1, Sq. C1, surface	*Hippopotamus* sp., tooth	27173	1257±29	-17.62	1060-1260
Taolambiby	Area 1, Sq. C0, 10-15 cm	*Cryptoprocta* cf. *ferox*, metacarpal	27174	1138±28	-17.86	936-1059
Taolambiby	Area 1, Sq. C0 5-10 cm	*Hippopotamus* sp., tooth	27175	1749±29	-19.32	1544-1702
Taolambiby	Area 1, Sq. C0 5-10 cm	Charcoal	27211	1121±26	-25.68	929-1056
Taolambiby	Area 1, Sq. C1	Charcoal	27212	1036±27	-24.18	803-957
Taolambiby	Area 1, Sq. A1, section 2	Charcoal	27213	1133±28	-24.49	934-1057
Taolambiby	Trench 2, Sq. 001	*Hippopotamus* sp., tooth	27305	1242±24	-17.12	1004-1182
Taolambiby	Area 1, Sq. C1	*Tenrec ecaudatu*, mandible	27306	1215±23	-18.28	983-1177
Taolambiby	OUM14346a/i	*Palaeopropithecus ingens*, tibia	26630	2112±26	-20.37	1935-2146
Taolambiby	OUM14346a/o	*Palaeopropithecus ingens*, humerus	26631	2445±27	-19.44	2346-2649
Taolambiby	OUM14346a/j	*Palaeopropithecus ingens*, radius	26632	2506±27	-20.3	2378-2711
Taolambiby	OUM14346a/l	*Palaeopropithecus ingens*, ulna	26633	2431±28	-19.65	2342-2683
Taolambiby	OUM14346a/c	*Palaeopropithecus ingens*, tibia	26634	2452±26	-19.67	2348-2696
Taolambiby	OUM14346a/a	*Palaeopropithecus ingens*, humerus	26635	2069±26	-21.00	1918-2056
Taolambiby	OUM14346a/	*Palaeopropithecus ingens*, radius	26636	2080±26	-20.99	1926-2082
Taolambiby	OUM14342a/A	*Palaeopropithecus ingens*, humerus	26637	2871±27	-20.16	2851-3057
